# Experimental and theoretical investigation of cationic-based fluorescent-tagged polyacrylate copolymers for improving oil recovery

**DOI:** 10.1038/s41598-024-78128-5

**Published:** 2024-11-12

**Authors:** Ali A. Abd-Elaal, Salah M. Tawfik, Ahmed Abd-Elhamid, Khalaf G. Salem, A. N. El-hoshoudy

**Affiliations:** 1 Petrochemicals Department, Egyptian Petroleum Research Institute, Naser City, Cairo, Egypt; 2 Department of Reservoir Engineering, South Valley Egyptian Petroleum Holding Company (GANOPE), Cairo, Egypt; 3 PVT Lab, Production Department, Egyptian Petroleum Research Institute, Naser City, Cairo, Egypt; 4 PVT-Service Center, Production Department, Egyptian Petroleum Research Institute, Naser City, Cairo, Egypt

**Keywords:** Enhanced oil recovery (EOR), Fluorescent polymers, Fluid flow, Simulation, Chemical engineering, Energy, Polymer chemistry

## Abstract

The growing need for energy and the depletion of oil wells necessitate advanced Enhanced Oil Recovery (EOR) techniques, particularly water and polymer flooding, which play a crucial role in augmenting hydrocarbon recovery rates. However, water flooding in high-permeability layers often leads to water breakthroughs, reduced sweep efficiency, and the formation of preferential channels, posing significant challenges to oil recovery and reservoir management. Conformance control treatments, including the use of polymer microspheres, offer a promising solution by sealing high-permeability zones and enhancing sweep efficiency. This study focuses on the application of fluorescent polymer microspheres based on polyacrylamide, which is extensively employed in the oil sector as an oil displacement agent. Fluorescent polymers called Poly 400, Poly 200, and Poly 600, incorporating cationic methacrylamide monomers, were synthesized through copolymerization to create amphiphilic polymers with enhanced stability and functionality. These fluorescent polymers were evaluated through flooding tests using a quarter-five-spot model of transparent quartz glass under UV light, allowing for instantaneous measurement and observation of fluorescence intensity. At reservoir conditions, the oil displacement experiments confirm that the incremental oil after water flooding by Poly 400, Poly 200, and Poly 600, is 13.1%, 9.1%, and 6.1% of OOIP respectively. The findings showed that fluorescent polymer microspheres could efficiently target high-permeability layers, adapt to varying pore throat sizes, and improve the plugging rate of high-permeability channels, thereby optimizing oil recovery. A subsequent simulation study using the CMG simulator provided further insights into the efficacy of these fluorescent polymers as EOR agents, revealing their potential to enhance sweep efficiency and enhance oil recovery. Simulation results showed that oil saturation decreased from 68% (initial) to 13.5%, 16.1%, and 18.3% after Poly 400, Poly 200, and Poly 600 flooding respectively. This work highlights the potential of fluorescent polymer microspheres as a valuable tool for EOR applications, offering significant advancements in reservoir management and oil recovery optimization.

**Supplementary Information:**

The online version contains supplementary material available at 10.1038/s41598-024-78128-5.

## Introduction

### Background

In the petroleum industry, flooding with water or polymers is an extremely important way to increase the amount of hydrocarbon recovery^1^, owing to increased energy demand, and depletion of the produced oil wells which require valorization of produced oil through chemical EOR techniques^2^. Water flooding in high-permeability layers often results in water breakthroughs, which decreases the pumped water’s movement performance and damages the pore structures, leading to the formation of larger pore throats. Additionally, these conditions can create "preferential channels," typically found in the space between the production and injection wells. The complexities of oil reservoir corrosion can negatively impact oil recovery, raise the cost of energy and eventually deteriorate environmental elements^3^. Conformance control treatments, such as injecting preformed particle gel, colloidal dispersion gels, weak gels, polymer microgels, biopolymer composite formulations, and polymer microspheres, can seal these high permeability zones^4,5^. This helps enhance the sweep efficiency of the injected water and ultimately improves oil recovery^6^. One of polymer microspheres’ benefits is that they can withstand extreme heat and salinity, as well as their capability to penetrate deep formations. As a result, polymer microspheres can improve sweep performance and successfully handle issues related to water erosion^3^. Some commonly used polymers in EOR, like polyacrylamide and partially hydrolyzed polyacrylamide, exhibit different ranges of temperature resistance^3^.

### Significance of polymer microspheres in EOR applications

Polymer microspheres consist of poly(ethylene glycol) (PEG) monomers encased by other vinyl monomers^7^. The addition of a fluorescent group enhances their properties, allowing them to serve as tracers for monitoring flow behavior in pipeline transportation and EOR applications. Fluorescence methods offer direct measurement and control of various treatment activities^8^. Usually, fluorescent substances like pyrene or naphthalene are used to label amphiphilic polymers that possess inherent fluorescence. For instance, pyrene-labeled short poly(ethylene oxide) chains can form micelles in water^9^. Fluorescence techniques provide versatile tools for monitoring solution properties with outstanding specificity and sensitivity, allowing the calculation of aggregation numbers in hydrophobic cores and critical aggregation concentrations^9^. Fluorescent polymers serve as convenient alternatives for tracking the growth, perspective, adsorption, and decomposition of polymers. They also help anticipate and evaluate the sweep ranges and oil displacement success by determining polymer concentration in generated liquid after flooding with polymers without requiring additional detection reagents or devices^2^. As tracers, fluorescent compounds provide quick, easy, and very sensitive fluorescence analysis, which makes precise assessment, automated detection, and management of water systems possible. Common fluorescent monomers, which are generally hydrophobic, can impact the structure of hydrophilic polymer microspheres, impacting their oil displacement efficiency, temperature and salt tolerance, conformity management, and swelling volume and duration^6^. Fluorescent nanoparticles like Ag NPs, FONs, and carbon QDs often suffer from aggregation, environmental toxicity due to leaching, and low stability. These issues can be addressed by using functional organic polymers with covalently bound fluorophore units, which improve stability and reduce toxicity risks^10^. In recent years, fluorescent polymers have been highly valued for sensing and imaging applications because they are easier to modify than small fluorescent compounds and have greater biocompatibility and water solubility^11^. These polymers are constructed by encapsulating fluorescent chromophores into a macromolecule matrix either through the modification of non-fluorescent polymers after polymerization or by straight polymerization using monomers or initiators containing chromophores^12,13^. The most common fluorophores include isothiocyanate (-NCS), active esters, free amine groups (–NH_2_), and carboxylate (-COOH)^12^. There are several polymerization processes used, such as atom transfer radical polymerization (ATRP), rapid addition-fragmentation chain transfer polymerization (RAFT), as well as free radical polymerization^11^. Fluorescent polymer microspheres achieve dual objectives: acting as conformance control agents and as indicators of oil deposits. Developing a fluorescent conformance control agent for oilfields is crucial. This agent can detect its concentration online and adjust it in real-time during the injection process, allowing precise research on its distribution within the formation^6^. Using fluorescent polymer microspheres as tracers in oilfields represents a novel application of fluorescent materials. They reveal information on circulation and routes by following the passage of injected fluids, such as water or polymeric solutions, inside the reservoir. This aids in evaluating fluid sweep performance by providing vital information on the rate with which injected fluids are dispersing oil within the reservoir, information that is crucial for maximizing EOR approaches. The performance of EOR approaches can be impacted by the data that fluorescent microsphere movement might disclose regarding reservoir variation, including differences in porosity and favored flow routes. The collection of precise data is made possible by the quantitative assessment of fluorescent microspheres, which helps determine the efficacy of the EOR operation and calculate recovery factors. Additionally, reservoir modeling and simulation outcomes can be validated using fluorescent microsphere tracking data to make sure they agree with expected findings^3^. The continual phase addition of fluorescent monomers to the mixture is the main characteristic of fluorescent polymer microspheres^3^. These microspheres are quite resilient and have no trouble penetrating the deeper reservoir layers. For efficient compliance control management, researchers highlight the significance of matching the size of polymer microspheres with pore throats^3^. By blocking high-permeability channels and allowing water to flow into low-permeability channels, polymer microspheres are primarily used to control excess water production and recover trapped oil leftovers^3^. While fluorescent polymers have been used in several industries, including medicine supply in biomedical applications^14^, scale inhibition, metal ion detection, CO_2_ and temperature sensing, and lipid droplet tracking, their application in the petroleum industry, to trace polymer sweeping during EOR operations, faces certain limitations.

### Literature review

Lu et al.^9^ prepared fluorescent amphiphilic copolymers, specifically polyacrylamide (PAM-b-PMATC), using the atom transfer radical polymerization (ATRP) method. Li et al.^15^ synthesized comb-like graft copolymers incorporating a fluorescent hydrophobic moiety. Wang et al.^13^ synthesized a novel fluorescent-tagged scale inhibitor to study the inhibition of scaling problems in cooling water systems. Huang et al.^16^ synthesized fluorescent organic nanoparticles (FONs) through RAFT polymerization and Schiff base combined^17,18^. Banerjee et al.^10^ developed a self-healing hydrogel with fluorescence activity by incorporating fluorescence-responsive ionic block copolymers (BCPs). Kang et al.^2^ prepared fluorescent polymeric materials by copolymerizing acrylamide (AM) with the chemically modified rhodamine B, for polymer flooding applications. Sand pack displacement experiments showed that the concentration curve of the fluorescent polymeric materials could be used through the displacement method to identify the breakthrough time and monitor concentration changes. Yang et al.^6^ developed a specialized class of fluorescent microspheres, P(AM-BA-RhB) polymers, as a novel approach to the conformance control process^3^. Oshchepkov et al.^19^ reported the preparation and characterization of a new fluorescent polyacrylate scale inhibitor. Yang et al.^6^ investigated the impact of incorporating various fluorescent functional monomers on the properties of the microspheres through their application in oil fields. Zhang et al.^11^ synthesized a fluorescent amphiphilic block polyacrylate copolymer using RAFT polymerization to investigate the interaction between lysosomes and lipid droplets, aiming to advance research in biomedical fields. Gao et al.^7^ provided a review of fluorescent microspheres, covering various synthetic techniques, characteristics, and applications. Liu and Zhang^1^ reviewed the use of phosphorus-derived polymers for potential uses in scale and corrosion inhibition within petroleum oilfields. Shagymgereyeva et al.^3^ reviewed the application of low-elastic and viscoelastic fluorescent nanocomposite microspheres for EOR in heterogeneous reservoirs. Table S1 (Supplementary materials) further summarizes the previous studies related to polymer microspheres.

### The gap and objectives of the work

The use of Fluorescent polymer microspheres for EOR remains a vibrant area of research, yet there are significant gaps and chances for additional research, particularly in developing formulations tailored to the distinctive qualities of various reservoirs^3^. The demand for fluorescent polymer microspheres in oil production is increasing due to their ability to provide real-time data on microsphere concentration and regulate their number during the injection process. These microspheres allow the analysis of produced fluid content and their proportion within it. Their robust structure, strong luminosity, and high environmental tolerance contribute to their growing success in the industry^3^. Upon entering a reservoir, these microspheres typically target high-permeability layers. Depending on their size relative to pore throats, they can either deform and pass through, block the entrance, or aggregate to form a barrier, thereby redirecting fluid flow and enhancing the blockage of high-permeability channels. This adaptability allows fluorescent polymer microspheres to penetrate deep formations and improve the plugging rate of these channels, ultimately optimizing oil recovery^3,20^. This work aims to explore new potential uses for fluorescent polymeric surfactants in oilfields. The research focuses on fluorescent polymers based on polyacrylamide, the most often utilized oil displacement agent in the industry. BCPs typically consist of at least one hydrophobic block and one hydrophilic block, resulting in amphiphilic polymers^12^. Flooding tests were carried out on a quarter five-spot model constructed from transparent quartz glass, which was subjected to UV light to facilitate the observation of fluid movement. This setup was connected to a sample collector, which was in turn linked to a spectrophotometer to measure the fluorescence intensity of the produced fluid. The primary objective was to monitor and quantify the concentration of fluorescent-tagged polyacrylate copolymers as they moved through the model, providing real-time data on their behavior and efficiency in the flooding process. The use of transparent quartz glass allowed for visual observation of the flow and distribution of the fluorescent-tagged polymers under UV light, enhancing the clarity and accuracy of the experiment. Subsequently, a simulation study was conducted at a lab scale using the CMG simulator to evaluate the performance of these fluorescent-tagged polyacrylate copolymers as potential Enhanced Oil Recovery (EOR) agents. Computer-aided simulation studies enable detailed investigation of processes at both atomistic and macro scales, providing precise and predictive insights into complex systems^21,22^. These simulations facilitate the modeling of physical, chemical, and petroleum processes, allowing researchers to explore scenarios and outcomes that are difficult or impossible to test experimentally^23^. By offering accurate predictions, these tools support the optimization of processes, enhance understanding of underlying mechanisms, and reduce the need for costly and time-consuming physical experiments^24^. The field-scale simulation helped in assessing their ability to improve sweep efficiency and increase oil recovery by modeling their interaction with reservoir rock and fluids under various conditions^25^. The combined experimental and simulation approach provided comprehensive insights into the feasibility and effectiveness of using fluorescent-tagged polymers for EOR, offering valuable data that could be extrapolated to field-scale applications.

## Experimental & methodology

### Materials & characterization

The used chemicals as summarized in Table 1 are of highest-grade chemicals obtained from Merck.

**Table 1:** Used chemicals and reagents.

Chemicals	Properties and purity %
Chloroacetic acid	~99%
Poly (ethylene glycol)	M_n~_ 200, 400, 600 Da
2-(Dimethylamino)ethyl methacrylate	~99%
N-[3-(Dimethylamino)propyl] methacrylamide	~99%
Acrylamide	AM ≥ 97%
Potassium persulfate	KPS ≥ 99%
Xylene	reagent grade
diethyl ether	≥ 99.7%
Toluene	≥ 99.5%
Petroleum ether	reagent grade
P-toluene sulfonic acid	97%

The synthesized polymers were analyzed using various spectroscopic methods. The infrared spectra were obtained using an American FTS-3000 spectrometer (400-4000 cm^-1^), with KBr discs employed to prepare the samples. ^1^H-NMR spectra were measured using a Bruker NMR spectrometer (400 MHz), with deuterium oxide used for solvation and tetramethylsilane (TMS) as a reference standard. Absorption spectra were recorded using a Jasco V-750 UV/Vis spectrophotometer (Jasco, Japan) in the measurement range of 200-600 nm, with a data interval of 1 nm and a bandwidth of 1.0 nm. Fluorescence spectra were collected using an Agilent Cary Eclipse fluorescence spectrophotometer (Agilent, USA, a quartz cuvette with a cm^-1^ path length). Rheological evaluations, including the examination of the shear viscosity and stress response as well as the viscoelastic characteristics, were performed on a concentric MCR 102e rheometer equipped with a cone-plate geometry^5^. To investigate the surface activity of the prepared cationic copolymer-based surfactants, the critical micelle concentration (CMC) was calculated using the electrical conductivity method at different concentrations using a portable multi-meter (HQ4100) with a conductivity probe (CDC401) at 25°C ± 1°C. The CMCs were extracted from specific conductivity-concentration plots as well and micellization free energy (∆G°_mic_) was determined using Equation  1^26^.

1 $$\Delta G_{mic}^{^\circ } = (2 - \beta )RT\ln C_{CMC}$$

where β, R, and T represent the degree of counter ion dissociation, the gas constant, and temperature, respectively, and C_CMC_ denotes the surfactant concentration.

### Synthesis of cationic methacrylamide derivative monomers

The synthesis of chloro-ester polyethylene glycol derivatives (Cl-PEG) involved a series of individual reactions. Chloroacetic acid and polyethylene glycol (with molecular weights of 200, 400, and 600) were equimolarly reacted in 100 mL of xylene. P-toluene sulfonic acid (0.01%) catalyst was introduced to the mixture. The mixture was refluxed until the required quantity of water (0.2 mol) was completely collected using a Dean-Stark apparatus. The resulting residue was then washed with diethyl ether to yield the chloro-ester polyethylene glycol derivatives (Cl-PEG200, 400, 600). Subsequently, in a series of individual reactions, a mixture of the synthesized Cl-PEG200, 400, 600, and methacrylamide derivatives (2-(dimethylamino)ethyl methacrylate and N-[3-(dimethylamino)propyl]methacrylamide) (0.05 mol) were heated in 100 mL of absolute ethanol for 24 h. Following evaporation of the ethanol, the prepared cationic polymer was purified with diethyl ether and recrystallization from ethanol, resulting in the formation of cationic methacrylamide derivative monomers labeled (E200, 400, 600) and (P200, 400, 600).

### Synthesis of cationic methacrylamide-co-acrylamide polymer

**Scheme 1:**
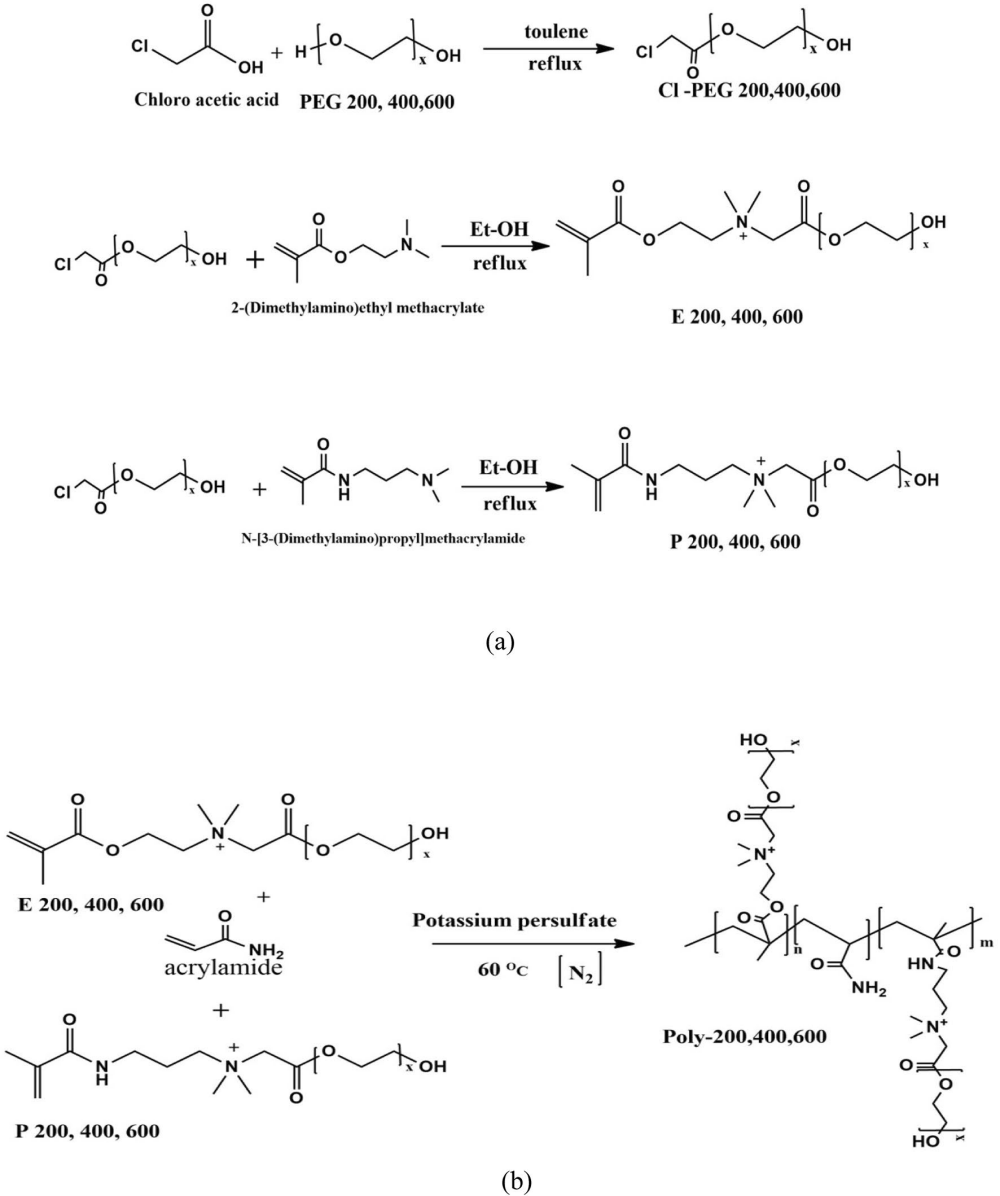
Synthetic routes of (**a**) cationic methacrylamide derivatives monomers; (**b**) synthetic route of cationic methacrylamide-co-acrylamide polymer (poly 600, 400, 200).

Dissolve 0.22 mol of acrylamide in distilled water within a 250 ml flask equipped with a condenser and thermometer. Sequentially add 0.011 mol of 2-(Dimethylamino)ethyl methacrylate and N-[3-(Dimethylamino)propyl]methacrylamide, followed by 0.0024 mol of KPS, stirring continuously under a nitrogen atmosphere for 20 min until a clear solution is formed. Set the temperature of the reaction to 59 °C and maintain it for 12 h to allow the polymerization to proceed via free-radical initiation. Cool the mixture afterwards, and induce precipitation of the gel using ethyl alcohol. The precipitate is then washed with acetone (3 × 50 ml), subjected to a 24-h Soxhlet extraction with petroleum ether at 50 °C, pulverized, and stored over a silica gel bed. Scheme 1a-b graphically presents the synthesis routes for these cationic methacrylamide derivative monomers and the resultant cationic methacrylamide-co-acrylamide polymer.

### Flooding and oil recovery

**Fig. 1:**
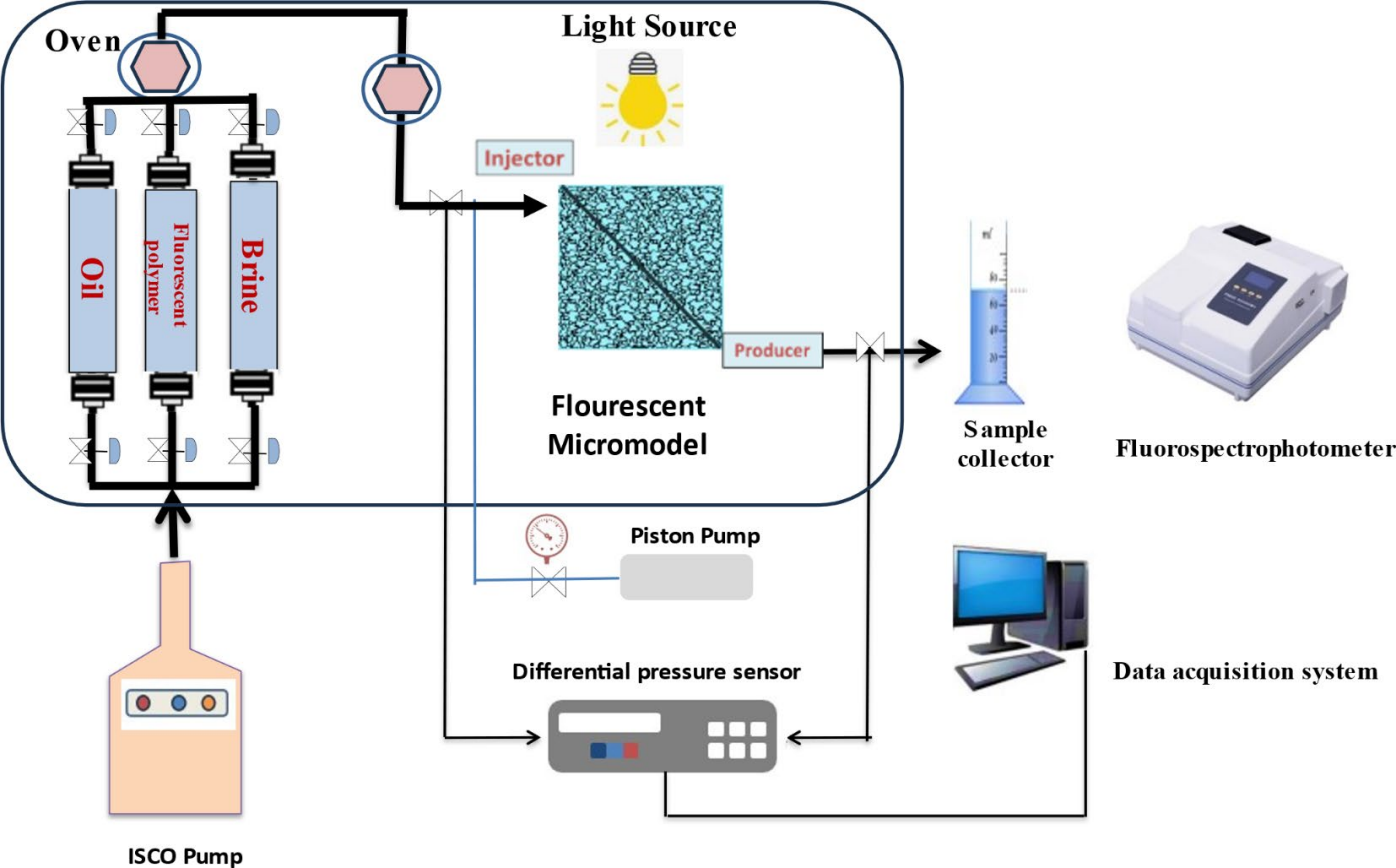
Schematic diagram of 1/4 five spot displacement model of fluorescent polymer.

Flooding experiments were conducted using a quarter (1/4) five-spot pattern model, constructed from transparent quartz glass with the dimensions of 30x30x5 cm. This model was filled with loose sand and equipped with an injector and a producer to simulate the comprehensive profile changes and the redistribution of oil in a mixed media, as shown in Figure 1. The model allows tracking the path of polymers, which glow under UV light due to their fluorescent properties, offering a fresh approach to using fluorescent polymers in enhanced oil recovery (EOR) approaches. A spectrophotometer is used to measure the fluorescence intensity of the produced fluid^2^. The sand was washed by standard procedures and then soaked in a 20,000 ppm NaCl solution for ten days before being saturated with oil. Following this, brine was injected at a rate of 3 ml/min until the oil cut was less than 1%. The porosity and volume of pores, along with the brine and oil’s relative permeability, were calculated using equations 2–6 cited in our prior work^27^. The differential in pressure and the injection velocity were monitored to observe the fluids’ movement and paths, alongside the fluorescence properties. Different three concentrations of fluorescent-tagged polyacrylate copolymers (1.0, 2.0, and 3.0 g/L) were injected at 90°C, followed by brine displacement until no more oil was produced. The total oil recovery was then measured against the injected pore volume.

2 $$Porosity(\Phi ) = \left( {\frac{Pore\;volume}{{Bulk\;volume}}} \right)$$

3 $$PoreVolume = \frac{{\left( {Wet\;Weight - Dry\;Weight} \right)}}{Formation\;Brine\;Density}$$

4 $$K = \frac{q\mu L}{{A\Delta P}}$$

5 $$K_{{rw}} = K_{w} /K$$

6 $$K_{{ro}} = K_{o} /K$$

## Results and discussions

### Spectroscopic analysis and characterization

**Fig. 2:**
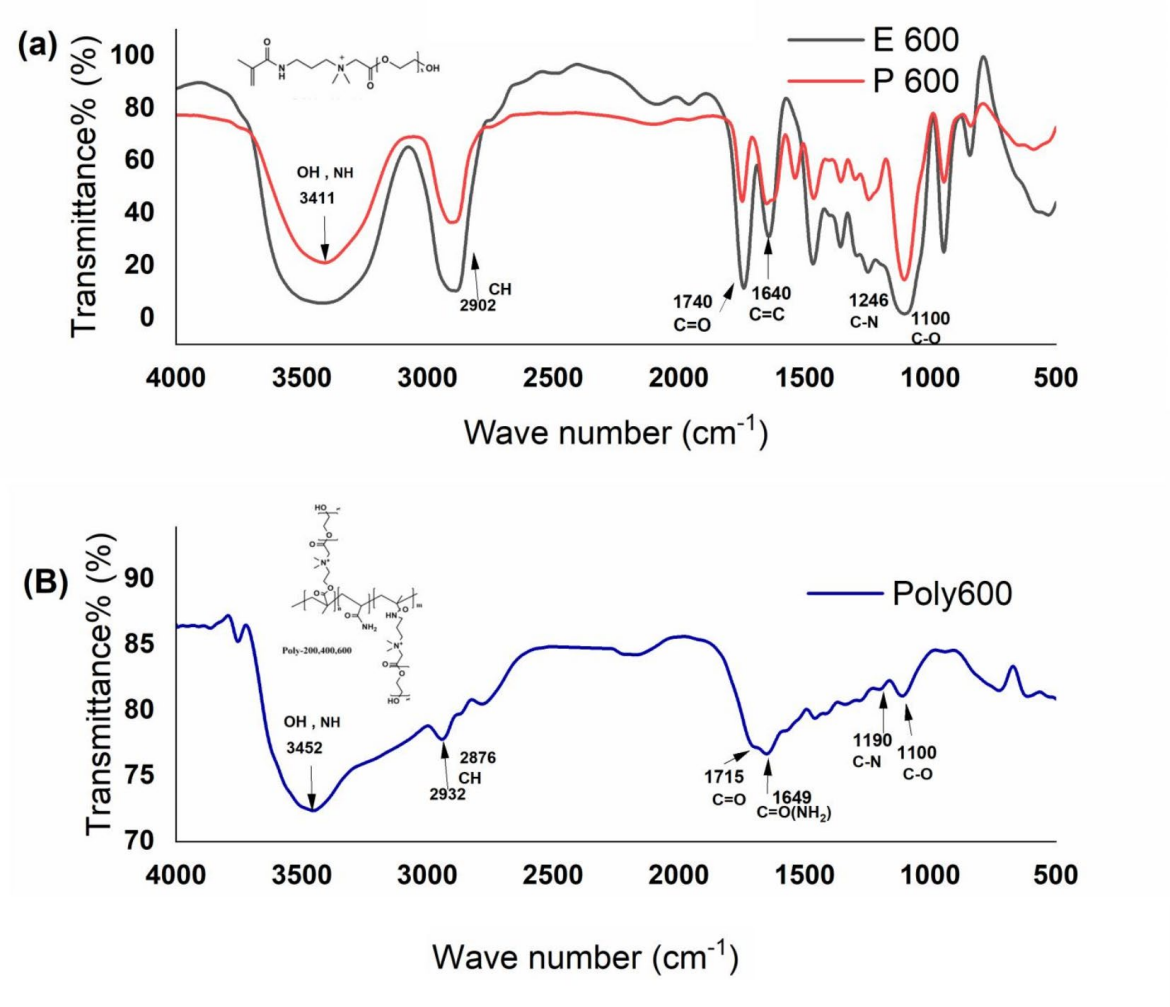
(**a**) FTIR spectra of cationic methacrylamide derivative monomers (P600 and E600), (**b**), represent FTIR spectra fluorescent polymer (poly600).

^**1**^**H-NMR spectrum of** fluorescent polymer (poly600) (Figure 3) show shifts at δ (a) = 1.1 (t, 3H, terminal –C**H**_**3**_ group), δ(b) = 1.46-1.92 (m, 26H, **CH**_**2**_CH_3_), δ (c) = 2-2.6 (t, H, NH_2_ -O = C-**CH-(**CH_2_)_2_, δ (d) = 2.9-3.1 (t, 2H, -O = C-NH**CH**_**2**_-CH_2_), δ (e) = 3.23- 3.5 (m, 3H, **(CH3)**_**2**_-N^+^-C**H**_**2**_**-**CH_2_), δ (f) = 3.85 (s, 3H, **CH**_**2**_-C(= O)-O-CH_2_), δ (ppm) = 7.19 (t, 2H, O = C-**NH**-CH_2_-) attributed to N, N-methylene bisacrylamide, and δ (ppm) = 6.83 (s, 2H, -N**H**_**2**_-C = O-) ascribed to acrylamide. Lack of a change in composition within the range of δ (ppm) = 5.63- 6.01 associated with vinyl bonds (-CH = CH_2_) in surfmer and other monomers verifies completion monomer polymerization^26^.

Figure 2a illustrates the FTIR spectra of cationic methacrylamide monomers (P600 and E600), alongside the fluorescent polymeric surfactant labeled as poly600. In Figure 2b, characteristic peaks corresponding to the cationic methacrylamide derivative monomers are apparent. At 2902 cm^-1^, the –CH_2_ group appeared, along with notable peaks at 1740 cm^-1^, indicating stretching vibrations of the C = O ester bond. Additionally, P600 and E600 monomers exhibit characteristic peaks at 3452 cm^-1^, ascribed to stretching vibrations of O-H, as well as peaks at 1246 cm^-1^ and 1100 cm^-1^, corresponding to C-O and C-N stretching vibrations, respectively. The distinctive peak at 1640 cm^-1^ represents the C = C bond. These findings confirm the successful preparation of the cationic methacrylamide derivative monomers. Figure 2b, represents a fluorescent polymer (poly600) resulting from the copolymerization of these monomers with acrylamide. The same distinctive peaks of the monomers were discovered with the addition of a peak at 1649 cm^-1^ corresponding to the C = O of the amide group in acrylamide. Notably, no signals related to C = C bonds are observed; indicating the disappearance of double bonds in the prepared monomers and declare occurring of the polymerization reaction. These distinctive peaks collectively signify the effective polymerization of the fluorescent polymeric surfactants.

**Fig. 3:**
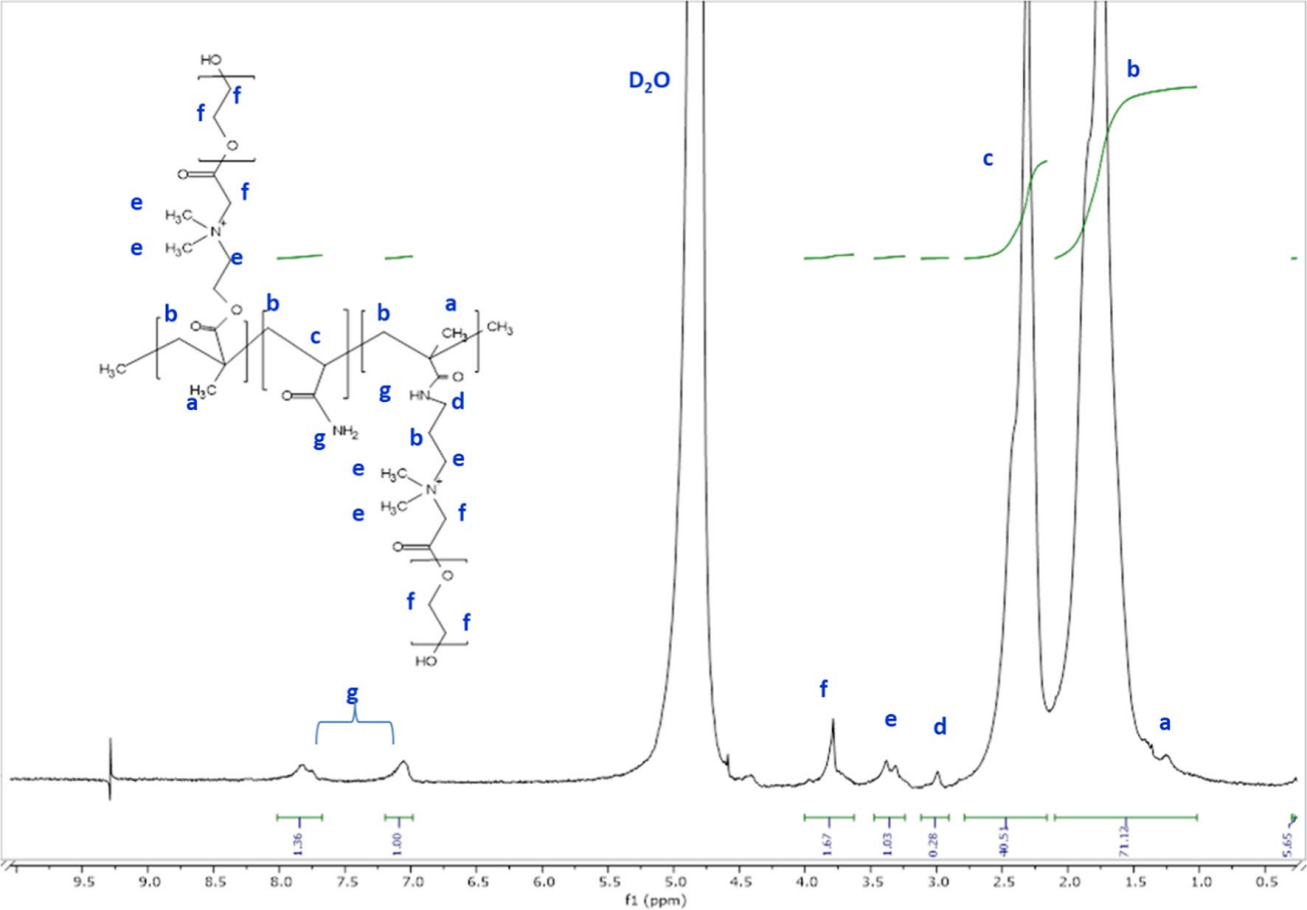
^1^H-NMR of fluorescent polymer Poly 600.

**Fig. 4:**
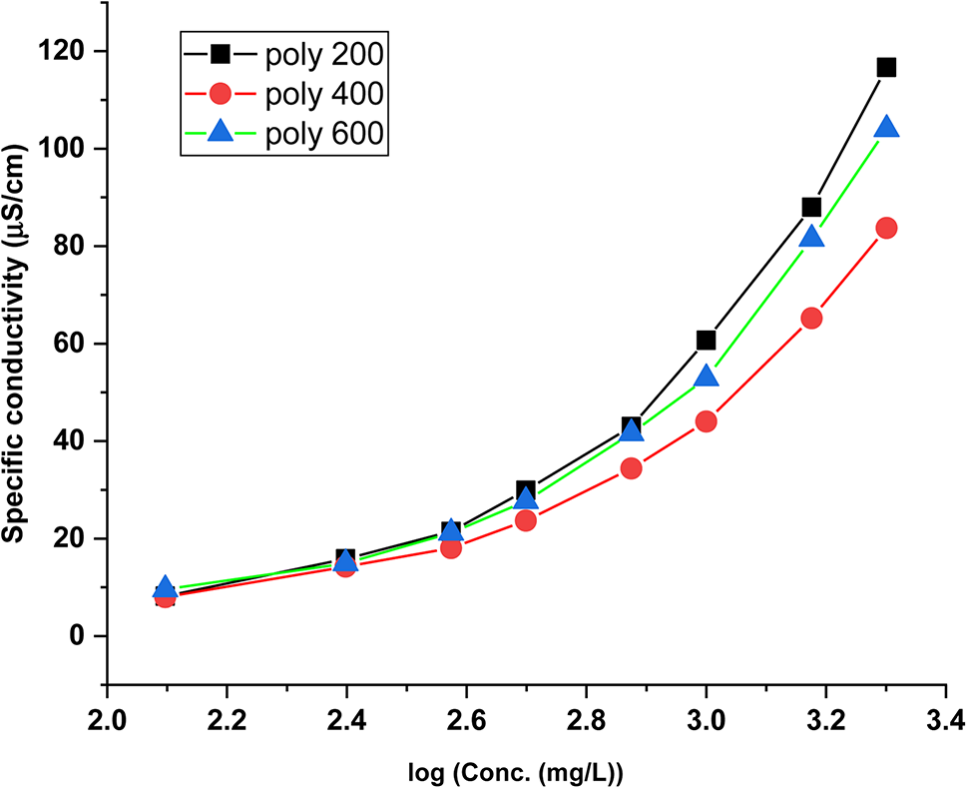
Specific conductivity against the logarithm of polymer concentration, plot for CMC determination.

The critical micelle concentration (CMC) of the synthesized copolymers in the aqueous media was determined using a conductivity test. Figure 4 displays the changes in conductivity values for poly 200, poly 400, and poly 600 polymeric surfactants at different concentrations. The intersection points in the specific conductivity (K) against copolymers concentration (C) curves were utilized to determine the CMC values of poly 200, poly 400, and poly 600 which were found to be 656.97, 716.45, and 691.63 mg/L. The counter ion dissociation degree (β) can be calculated from the ratio of the two slopes. The micellization free energies (∆G^o^_mic_) based on CMC values were determined using Eq. (1). The obtained micellization of free energy (∆G^o^_mic_) of the prepared poly 200, poly 400, and poly 600 are negative – 27.95, –26.16 and –24.07 kJ mol^−1^, respectively, suggesting that the micellization phenomena is favorable and spontaneous in expressions of decreasing the solution energy due to the micelle dissolution in aqueous medium^28^.

### Optical properties and UV spectroscopy

**Fig. 5:**
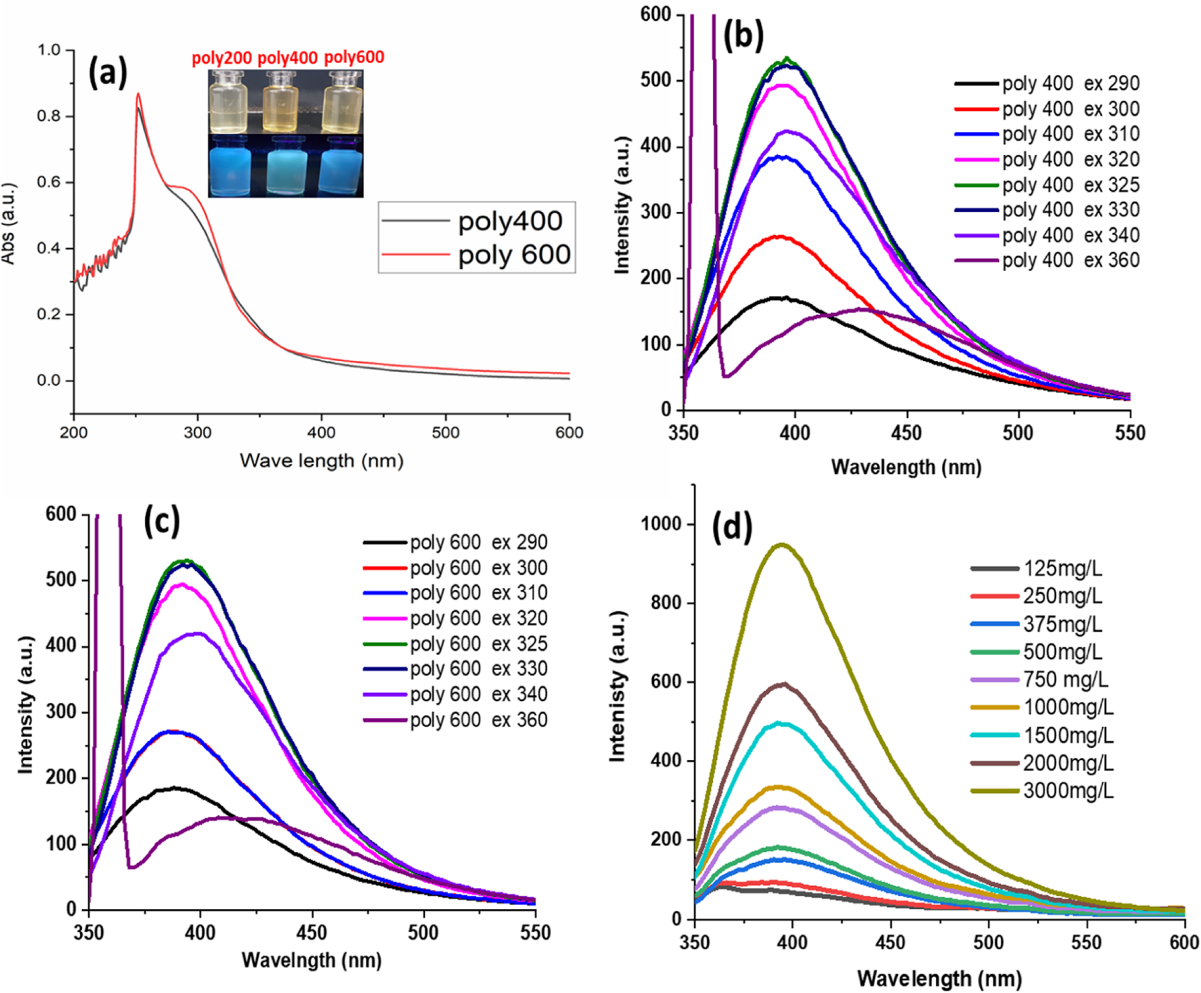
(**a**) Absorption spectra of P400, and P600. (**b**,**c**) Fluorescence spectra of P400, and P600 at various excitation wavelengths in water. (**d**) The changes of fluorescence intensity of P400 with different concentrations.

UV–vis absorption and fluorescence studies were used to examine the optical characteristics of the P400 and P600. Figure 5 illustrates that the electron transitions of n-π* for C═O and n-π for C═N bonds are attributed to a large absorption peak located at 340 nm, respectively. In the meantime, when exposed to 365 nm UV light, the P400, and P600 exhibit an intense blue color emission (inset of Fig. 1). The fluorescence emission spectra of P400 and P600 were measured and are presented in Fig. 5 and the maximum emission (λ_em_) peaks are 395 nm, and 393 nm, respectively. Furthermore, the polymers display excitation-dependent emission behavior when the excitation wavelength is altered from 290 to 360 nm. The uneven defects in the surface are responsible for the slight redshift seen in Figure 5 for the emission peak. Furthermore, Figure 5 illustrates the relationship between the fluorescent polymer’s intensity and polymer concentration, showing that intensity improved as polymer concentration increased.

### Effect of pH, Temperature, and ionic strengths on fluorescence intensity

**Fig. 6:**
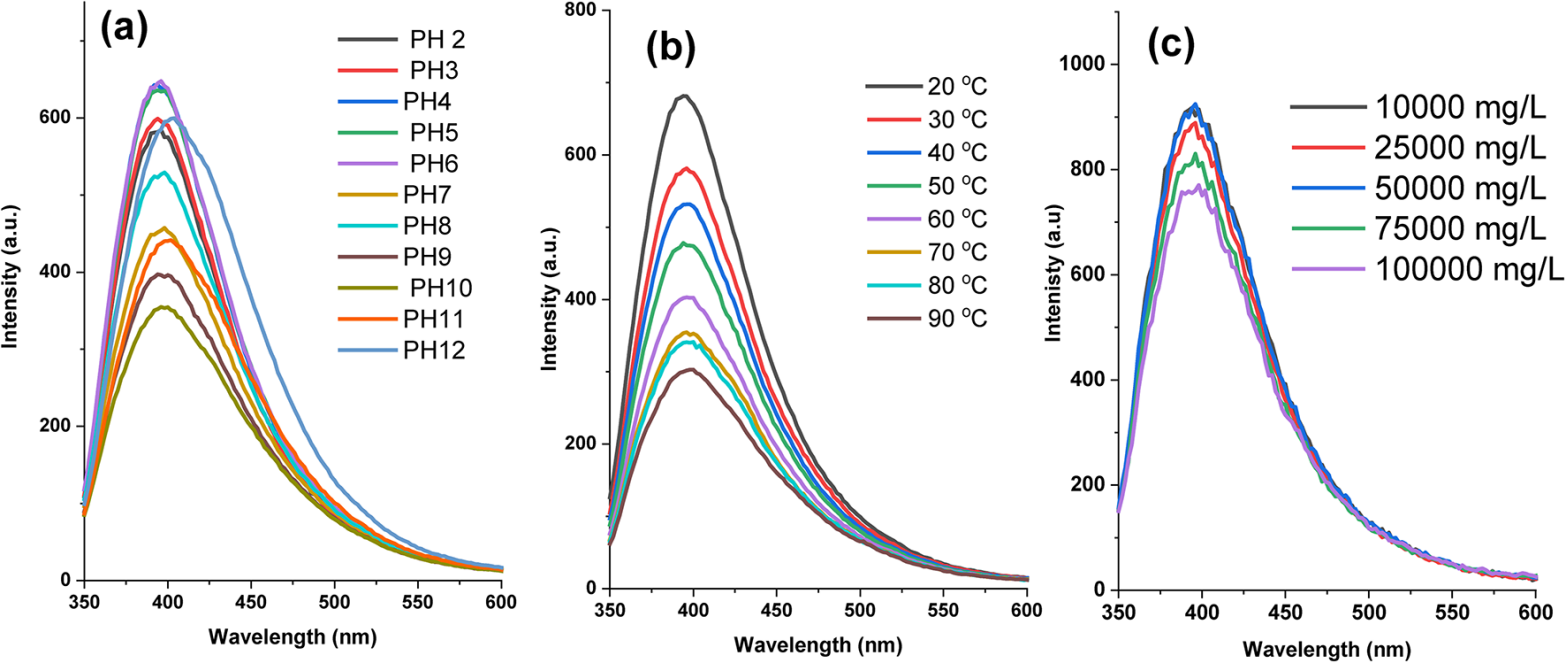
The effects of (**a**) pH, (**b**) temperature, and (**c**) ionic strengths on fluorescence spectra.

To study the fluorescence changes of the synthesized P400 with pH levels steadily varying from 2 to 12, the fluorescence spectra of P400 distributed in various pH value solutions are assessed using a spectrophotometer as shown in Figure 6. Figure 6a shows the fluorescence spectra of the fluorescent polymers to the pH value. P400 presented similar pH stability trends and the high FL intensity displayed in a pH range of 2-7 a comparable stability with 34%, and 32% a drop of initial fluorescence intensity at pH 9 and 10, respectively. The phenomenon under investigation could perhaps be attributed to alterations in the electron cloud density of fluorescent monomers and the diminution of conjugation cationic structure. As a result, the system’s fluorescence intensity decreases. Furthermore, Figure 6b illustrates the correlation between temperature and fluorescence intensity. The intensity of the fluorescence gradually decreased as the temperature increased. This phenomenon’s main cause is explained as follows: when the temperature rises, molecules move faster, increasing the possibility of intermolecular collisions and, consequently, the non-radiative transition. Thereby fluorescence efficiency and fluorescence intensity decrease. In addition to the pH and temperature stability, we investigated the behavior of the fluorescent polymers in aqueous solutions at various ionic strengths, as shown in Figure 6c. Over the concentration range of NaCl used (1000-10,000 ppm), little or no change was observed in FL intensity compared to blank (0 M NaCl). These experiments further highlight the stability of these new polymers in high ionic strength conditions.

### Rheological characteristics and viscoelasticity

#### Shear/viscosity and Stress scanning

**Fig. 7:**
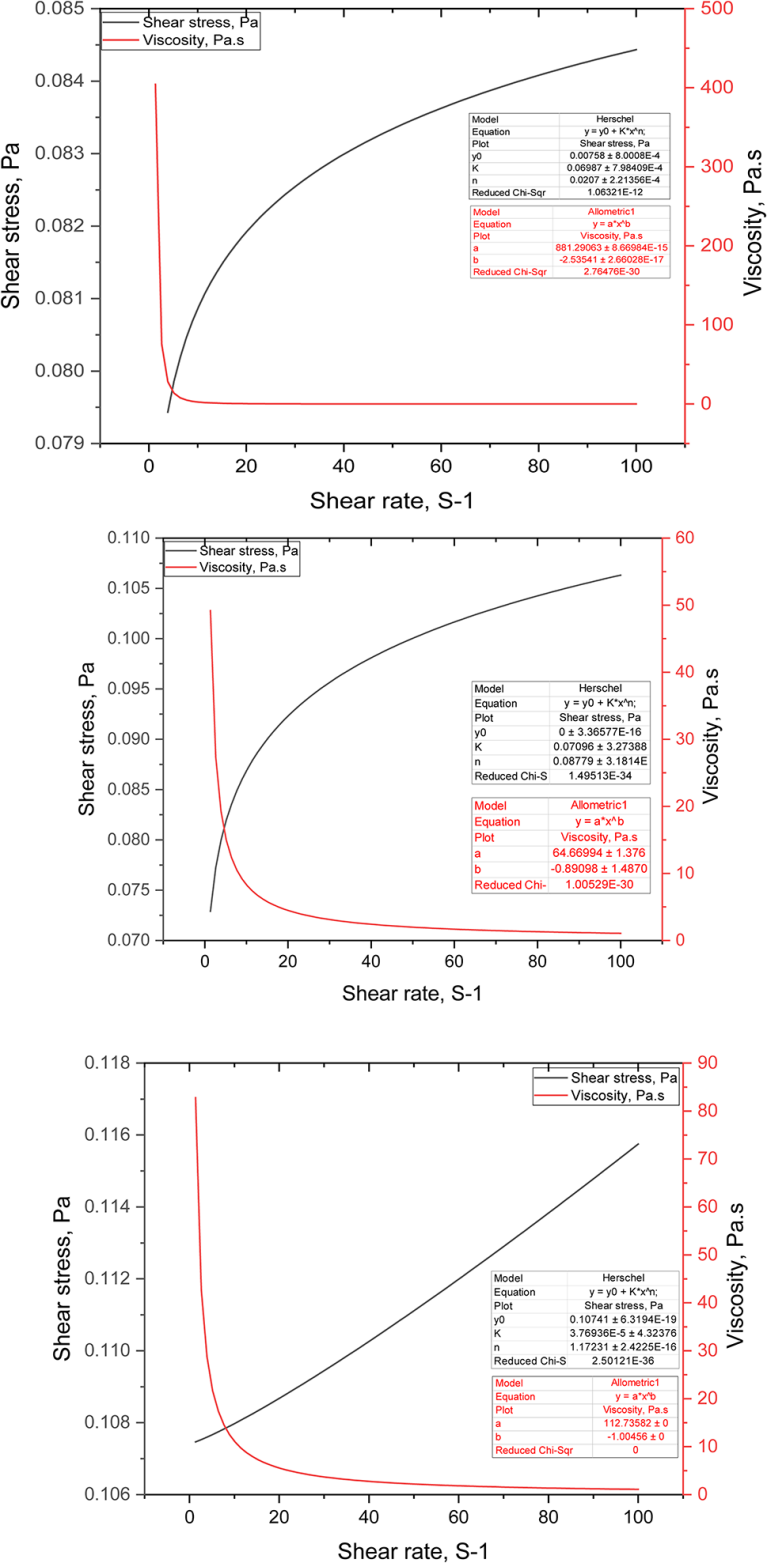
Shear/viscosity profile and stress scanning of poly 200, 400, and 600.

Shear rate significantly impacts the phase morphologies of methacrylamide-co-acrylamide copolymers^28^. In this regard, the shear/viscosity profiles and stress responses of the samples were assessed over a broad shear rate range at a temperature of 90°C, as illustrated in Figure 7. The shear stress and shear rate data conformed to the Herschel-Bulkley model, a conventional flow equation^29,30^. Meanwhile, the shear rate and viscosity (µ) were described by the Allometric power law (Equations. 7 & 8). The raw dataset can be found in the supplementary material (Figure S1). The prepared polymer solutions (Poly 200, 400) exhibit shear-thinning behavior, characteristic of pseudoplastic fluids where n ≤ 1.0^31^. This property is advantageous for use as chemical flooding agents because it allows the polymers to maintain viscosity under shear^32^. However, Poly 200 and Poly 400 show very low initial yield stresses, suggesting easy flowability of the suspensions. Specifically, the yield stress is nearly zero for P400. Low yield stress implies that minimal stress is required to initiate flow in the polymers, indicating that no significant shearing is necessary for their movement This characteristic suggests that polymers can move with ease even under low applied forces, highlighting their ability to flow without the need for extensive shearing^30,33,34^.

7 $$\tau = \tau 0 + K\gamma^{n}$$

8 $$\mu = K\gamma^{ - n}$$

Figure 7 shows that Poly 200 and 400 closely follow the Herschel-Bulkley model, indicating pseudoplastic behavior with yield stress; this is evidenced by values of (n) < 1.0 as presented in Table 2. These findings confirm that the tested samples are pseudoplastic (viscoelastic) fluids, making them suitable for polymeric displacement processes^31,32^. The shear-thinning performance is attributed to the progressive molecular entanglements reduction as the shear rate increases^35^. The shear-thinning behavior would be advantageous, especially when handling or transporting polymer at the surface before injecting it into the reservoir pores^36^. This property is beneficial during the injection of EOR fluids into reservoirs as it reduces the energy requirements for pumping, making operations more efficient. With increased shearing, cross-links break down leading to a reduction in system flow resistance due to dehydration, thereby decreasing viscosity^37^. As a result, the gel structure deteriorates under high shear conditions, flowing through narrow pores with minimal rupture and re-forming upon resting in the pores^38,39^. Conversely, P600 shows an (n) value greater than 1, specifically 1.17, which might suggest a deviation towards shear-thickening behavior. However, the shear viscosity curve reveals that P600 still exhibits shear-thinning properties as viscosity decreases with increased shear. This unusual behavior resorts to the complex molecular weight of the monomers E600, and P600 in Poly600, initially suggesting shear-thickening. Nevertheless, viscosity decreases consistently with increased shearing up to 40.0 S^-1^ and then slightly levels off. This behavior can be linked to hydrophobic and hydrogen bonding interactions within the molecules^26,40^.

**Table 2:** Data analysis of shear/viscosity and shear/stress profiles.

Parameters	(Herschel-Bulkley model)			(Allometric power law model)		
	P_200_	P_400_	P_600_	P_200_	P_400_	P_600_
τ_0_, Pa	0.0075	0.0	0.1074	N/A		
K, Pa. s^-n^	0.0698	0.0709	3.76E-5	881.2	64.66	112.7
n	0.0207	0.0877	1.17	-2.535	-0.8909	-1.00

The cationic-based fluorescent-tagged polyacrylate copolymers demonstrated heat resilience due to the 3D structure within the polymer microspheres. Raising the temperature from 25°C to 90°C significantly affected their swelling properties. The fluorescent-tagged polyacrylate copolymers offer advantages such as good elasticity, strong resilience to harsh reservoir conditions, and effective plugging performance^3^.

#### Viscoelastic properties

Dynamic frequency sweep tests were utilized to explore the network formation and microstructure within the nanocomposites in the linear viscoelastic region (LVR). Additionally, these tests evaluated the viscoelastic properties of the samples over time^41^. Viscous fluids do not exhibit elastic deformation when exposed to shear stress; but, they dissipate the applied force and energy as heat. In contrast, viscoelastic systems possess both elastic and viscous properties. The dominant behavior either elastic or viscous depends on the duration over which stress is applied. Viscoelastic properties are determined by measuring the shearing moduli, which include the viscous modulus (G″) and the elastic modulus (G′). The elastic modulus, also known as the storage modulus or dynamic rigidity, quantifies the energy stored reversibly as deformation^42^. On the other hand, the viscous modulus, or loss modulus, reflects the energy irreversibly lost as heat during a cycle. These measurements are conducted at a constant temperature (90°C) and an angular frequency ranging from 1 to 100 rad/s, with a strain set at 0.05^43,44^. This setup ensures that the oscillatory deformation remains within the linear viscoelastic range^30,45,46^. The viscoelasticity of materials is evaluated by measuring the stress response to a sinusoidally oscillating shear strain. This shear stress is calculated using the torque according to Equation 9^47^:

9 $$\tau = \tau 0\sin (\omega t + \delta )$$

Furthermore, the relationship between various moduli and the phase angle is described as a function of (G′), (G″), (G*), and (δ) as formulated in Equations 10, and 11^38^.

10 $$G^{\prime} = G*\cos \delta$$

11 $$G^{\prime\prime} = G*\sin \delta$$

**Fig. 8:**
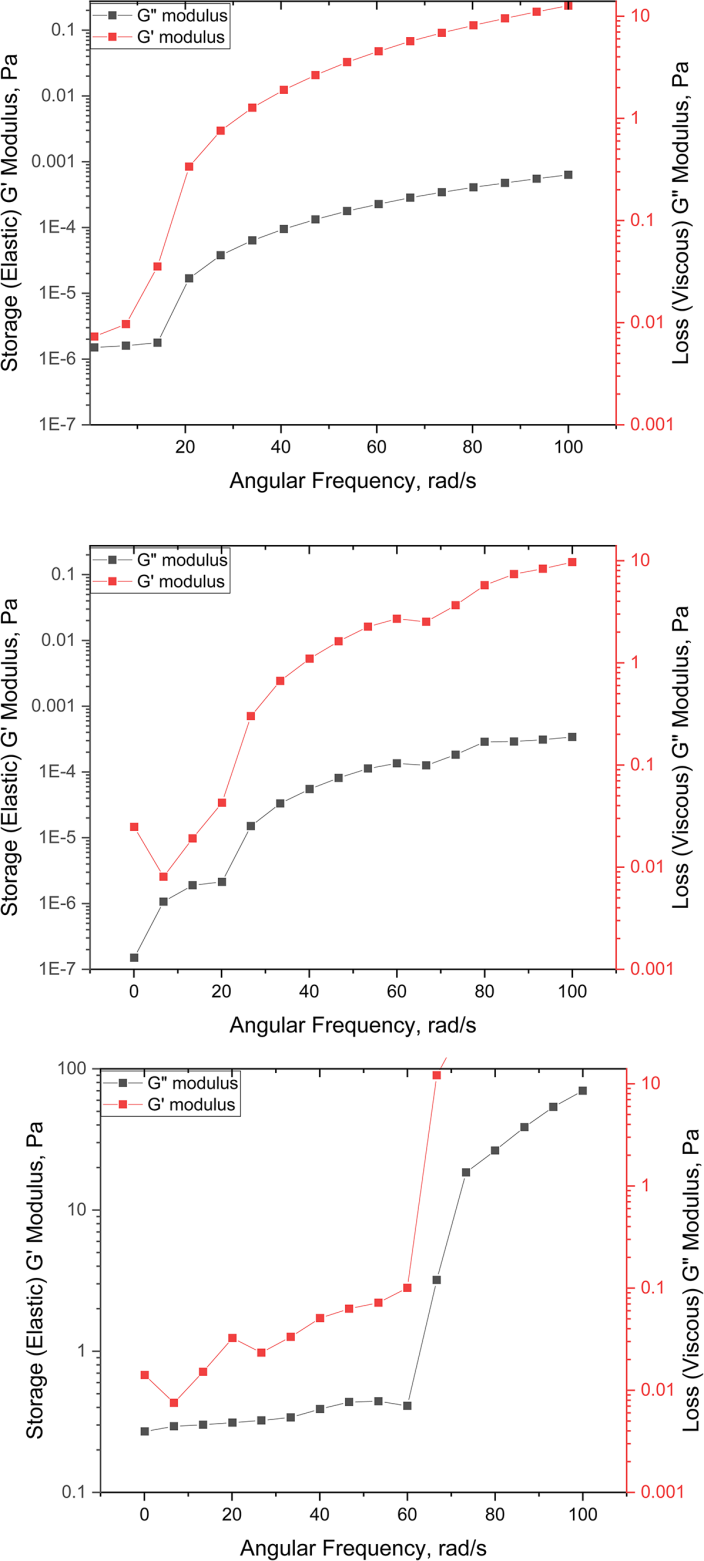
Viscoelastic properties of Poly 200, 400, 600 respectively.

Figure 8 indicates that the (G′) is greater than the (G″), suggesting that the hydrogel exhibits typical gel-like characteristics^44^. A higher elastic modulus implies that the material has a greater capacity for energy storage. Consequently, materials with a higher elastic modulus are likely to recover their original shape more effectively after the removal of applied force^5^. This enhanced viscoelasticity resorted to the polymer’s crosslinked network structure and the formation of hydrogen bonds, which collectively improve the material’s elastic properties.

## Estimation of oil recovery

**Fig. 9:**
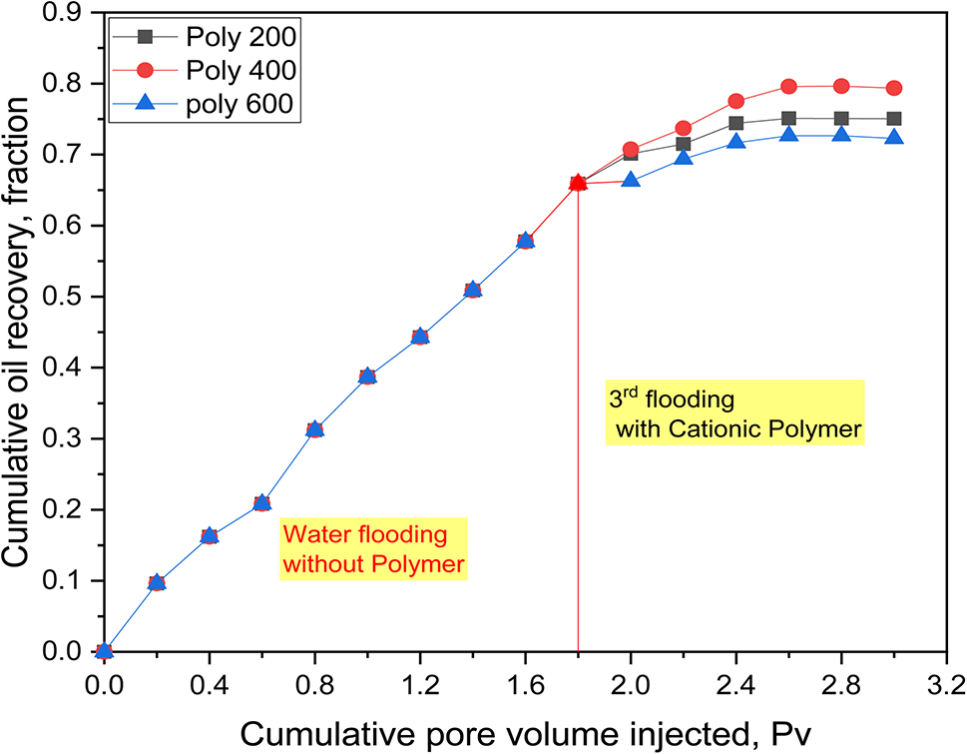
Cumulative oil recovery with fluorescent cationic polymers.

Polymer overflowing is designed to raise the viscosity of the water phase, thereby improving sweep efficiency^48^. In this experiment, displacement tests were performed using a 1/4 five-spot model. Fluorescent cationic polyacrylamide polymers at a concentration of 2000 ppm were employed, and the polymer flow was observed using a UV lamp through a glass 1/4 five-spot model. The results, as shown in Figure 9, indicate that Poly 400 achieved the highest recovery factor. The recovery factors increased in the following order: Poly 400 (79% OOIP), Poly 200 (75% OOIP), and Poly 600 (72% OOIP), demonstrating successive improvements in oil recovery. The cumulative oil recovery from water flooding alone reached 65.9% after the injection of 1.8 pore volumes (PV). This recovery rate was further enhanced with the addition of cationic-based fluorescent-tagged polyacrylate copolymers. Therefore, the incremental oil after water flooding by Poly 400, Poly 200, and Poly 600, is 13.1%, 9.1%, and 6.1% of OOIP respectively. The ability of the prepared copolymers to increase the recovery factor is attributed to their capacity to form micelles around oil globules, facilitated by the presence of PEG moieties. Additionally, these polymers increase the viscosity of the displaced fluids, thereby improving sweep efficiency^49–51^. It should be highlighted that cationic-based fluorescent-tagged polyacrylate copolymers reduced the mobility ratio (M) between the aqueous and oleic phases from 14 in the case of water flooding to less than unity in the case of polymer and thus improved the sweeping efficiency. According to Figure 9, Poly 400 achieves higher oil recovery rates than Poly 200. This improvement is due to the increased molecular entities in Poly 400 (P400, E400), which can solubilize more oil and enhance sweeping efficiency. In contrast, the recovery factor for Poly 600 is lower. This decrease is attributed to the enhanced surface-active properties of Poly 600 (P600, E600) compared to Poly 200 and 400, leading to the formation of (O/W) emulsions. These emulsions obstruct oil flow due to high-pressure differentials (ΔP) across the sandpack model, ultimately reducing oil permeability and recovery^40,52^. Moreover, while a small number of surface-active moieties stabilizes hydrophobic associations, increasing their concentration results in the encapsulation of hydrophobic groups and association disruption. Consequently, the viscosity of the solution decreases, further diminishing the amount of oil recovered^27^.

It should be highlighted that the combined effect of the polymer’s amphiphilic nature and ionic charge results in a significant reduction of interfacial tension between the oil and water phases. The integration of PEG and cationic surfactants in modified polymers creates a powerful synergy that significantly enhances oil displacement and recovery processes. These innovative polymers possess an amphiphilic nature, allowing them to interact seamlessly with both aqueous and oil phases within reservoir rocks. The PEG backbone contributes to the polymer’s water solubility and stability in challenging reservoir conditions, while the hydrophobic components of the cationic surfactants engage with oil droplets. This dual affinity facilitates the formation of stable oil-in-water emulsions, promoting the mobilization and transport of trapped oil through porous media. The positively charged groups in these polymers play a crucial role in their effectiveness. They interact electrostatically with negatively charged rock surfaces, minimizing polymer adsorption onto the reservoir rock and improving its propagation throughout the formation. Additionally, these cationic charges aid in altering the rock surface wettability from oil-wet to water-wet, a critical factor in enhancing oil recovery efficiency. The combined effect of the polymer’s amphiphilic nature and ionic charge results in a significant reduction of interfacial tension between the oil and water phases. This reduction is vital for mobilizing residual oil trapped in small pores and crevices within the reservoir rock. Furthermore, the cationic groups enhance the polymer’s ability to form viscoelastic solutions, leading to improved sweep efficiency and more effective oil displacement^53^.

## Adsorption retention and pressure variation during displacement

**Fig. 10:**
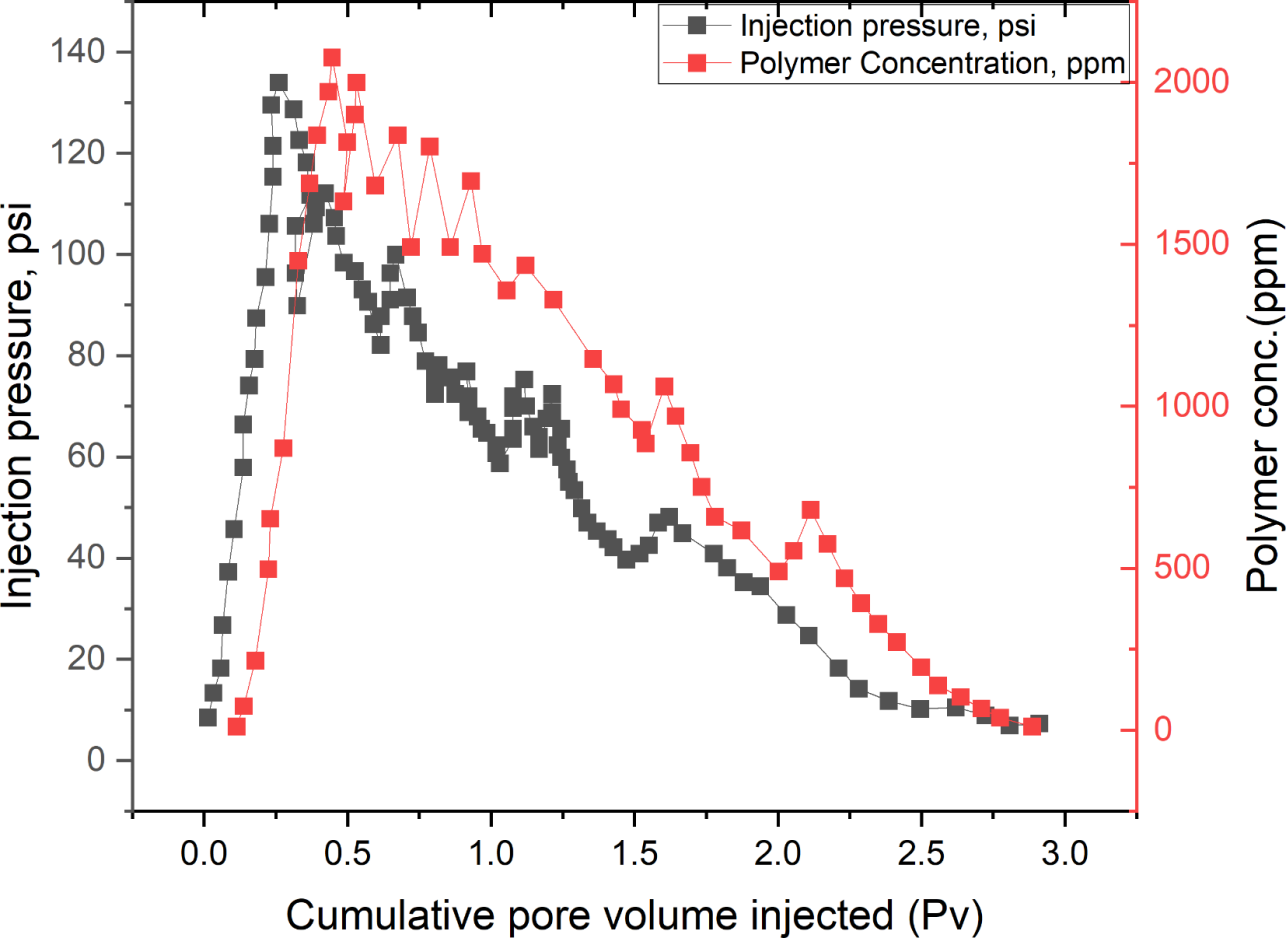
Adsorption Retention and Pressure variation as a function of (Pv).

Adsorption retention refers to the ability of the sand pack’s porous media to hold onto the polymer molecules. As the polymer is injected, it interacts with the sand, and some of the polymer is retained through adsorption. This is a crucial factor because it affects how much of the injected polymer is ejected to the outlet. The diminishing polymer concentration observed could be indicative of high adsorption retention within the sand pack. This adsorption impacts both the flow dynamics and the concentration of polymer detected at the outlet. The pressure variations observed during the injection process provide insights into the physical interactions within the sand pack. Initially, the pressure increases as the injection volume rises, reaching a peak at 0.25 PV due to the resistance against the flow of polymer through the porous media. This peak pressure might also reflect the point of maximum resistance or clogging within the sandpack, where the pores are getting saturated with polymer. When the polymer begins to be detected at the outlet at 0.45 PV and a concentration of 2000 ppm, the subsequent pressure fluctuations and gradual decrease can be attributed to the varying levels of blockage and release within the sandpack as shown in Figure 10. These fluctuations are typically caused by the non-uniform distribution of the polymer, the possible formation of filter cakes, or temporary plugging by polymer aggregates. The concurrent fluctuations of the injection pressure and polymer concentration can be analyzed to understand the percolation state of the polymer through the sand pack. The pressure drops accompanying the detection of the polymer at the outlet suggest a breakthrough, followed by the easing of some initial resistance or blockages within the sand. The fluctuations in polymer concentration could also point to varying flow paths or channels forming within the sand pack, altering the flow dynamics and concentration profiles. Combining the pressure and concentration curves provides a detailed view of the polymer’s behavior during the displacement process. This analysis is crucial for determining:

- Breakthrough time: When the polymer first appears at the outlet.
- Concentration variations: How polymer concentration changes during the process indicates the efficiency and effectiveness of the displacement.
- Percolation state: Understanding how well the polymer navigates through the sand pack, can inform adjustments in injection strategies to optimize recovery or filtration processes.

The use of fluorescent polymer concentration as a detection method appears effective for monitoring the dynamics within the sand pack. The analysis of pressure and concentration fluctuations not only aids in understanding the percolation and adsorption characteristics but also provides essential data for improving polymer injection strategies in similar geological or industrial applications^2^.

## Core-scale simulation

**Fig. 11:**
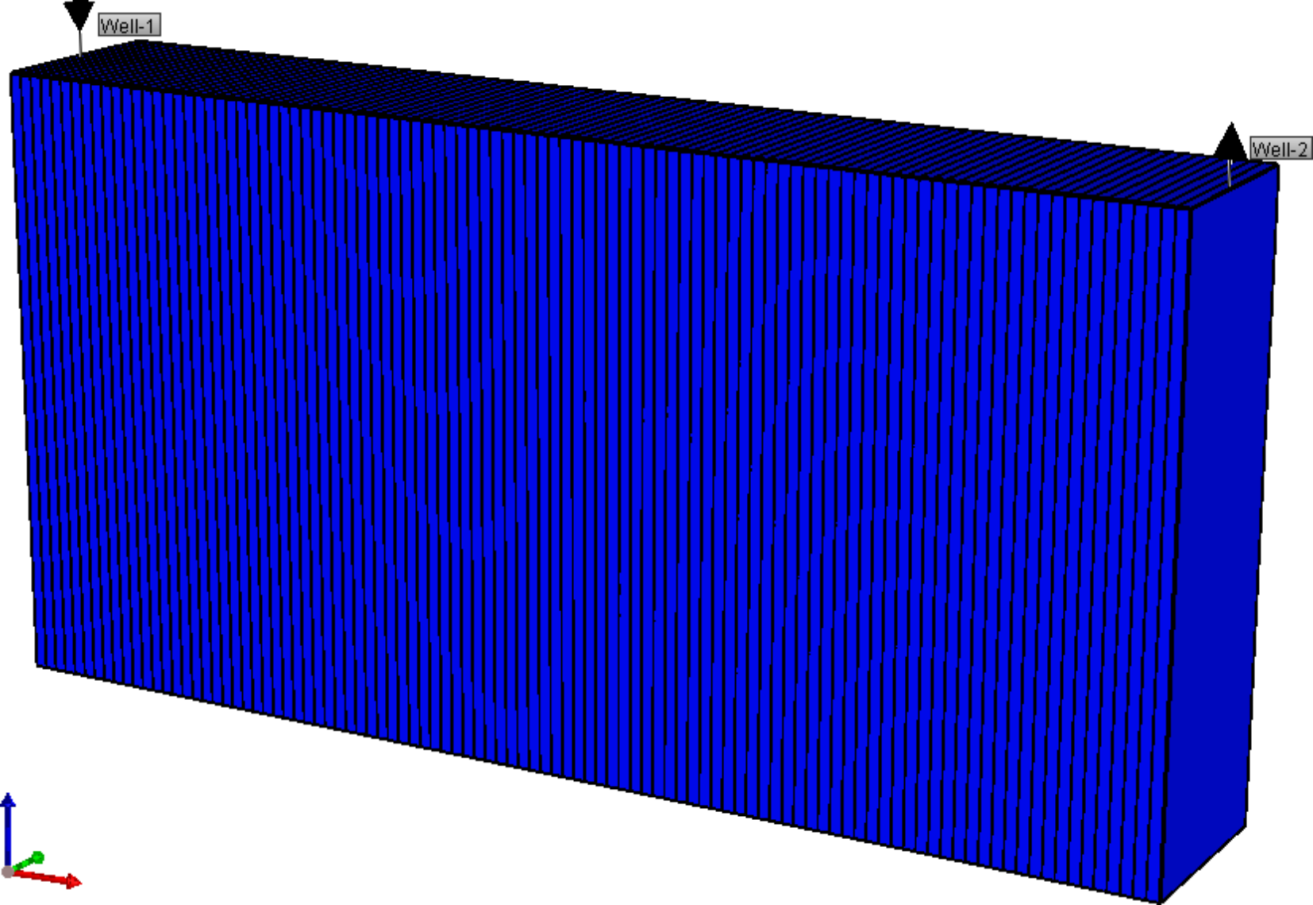
Cartesian core flood simulation model.

CMG STARS simulator is employed to validate the polymer flooding experiments at the core and field scales^54,55^. A Cartesian rectangular grid was used to create the core flooding runs as shown in Figure 11. One hundred blocks in the I direction were allocated to enhance the simulation’s correctness. The block number in the j and k directions = 1.0 to simulate a one-dimensional flow^56^. Each grid length was set equal to 0. 3 cm in I-direction. Height and width were set to 5 cm to equal the bulk volume of the core scale. The porosity, absolute permeability, and initial oil saturation values were 35%, 850 md, and 68% respectively. Table 3 presents the specifics of the core model and fluid properties used in the simulation. The technique of history matching is employed to simulate and predict the outcomes of chemical flooding^54,56^. A primary objective of this section is to perform history matching of core flooding experiments and to forecast the outcomes under conditions that were not feasible to execute in the laboratory. The injector well was established at the cell (1 1 1), while the producer well was placed at (100 1 1) as stated in Figure 11. Taking into consideration, that the well-bore radius for both injector and producer wells was equal to 0.3 cm in K-axis. Datasets concerning the pore volume of the formation, grid formation, initial saturation, volume of the oil phase, and oil viscosity are displayed in Table 3. The oil recovery and oil saturation parameters were chosen for history matching.

**Table 3:** The core, fluid, and polymer properties in the CMG STARS Simulator.

Type of data	Property	Value
Core and fluids properties	Core type	sandstone
	Core length, cm	30
	Core diameter, cm	5
	pore volume, cm^3^	320
	Porosity, %	35
	Permeability, mD	850
	oil gravity, API	34
	oil viscosity, cp	5
	water viscosity	0.3
Relative permeability Data	kro	0.45
	krw	0.17
	Sw_i_,_%_	32
	so_r_	17.4
Polymer properties	Polymer adsorption, mg/g	50
	RRF	5
	IPV, %	10
	polymer concentration, ppm	2000
Wells constraints	Injection rate, cc/min	0.5
	production rate, cc/min	0.5

**Fig. 12:**
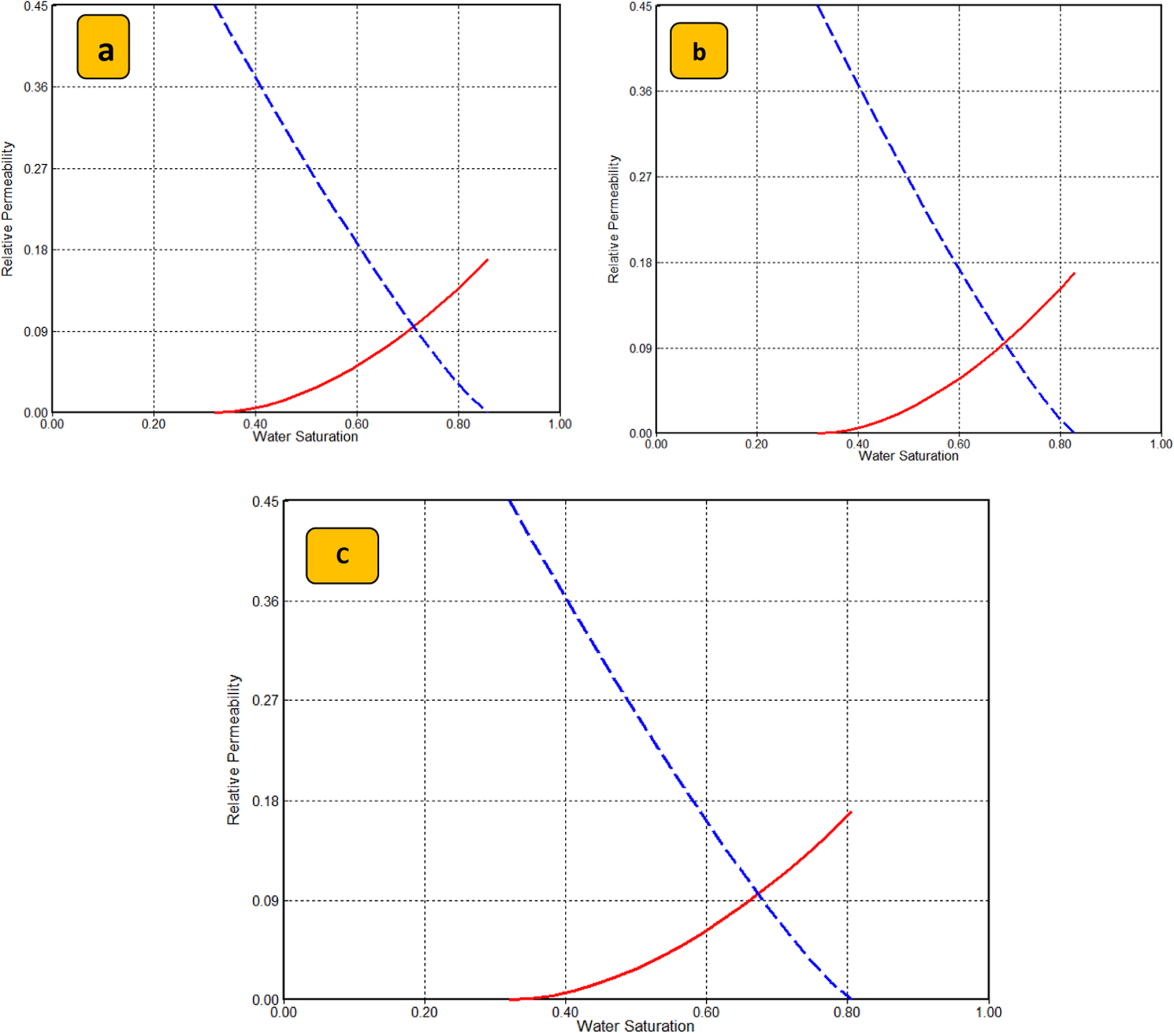
Modeling of relative permeability using CMG STARS (**a**) Poly400 (**b**) Poly200 (**c**) Poly 600.

More importantly, modifying and tuning the relative permeability curves was a crucial aspect of accomplishing a good history match for the flooding during the EOR process^57–59^. Combining the experimental data measurement of relative permeability with Corey’s correlations defaulted in the CMG STARS simulator to model the relative permeability curves as depicted in Figure 12. Using CMG-STARS, Corey’s correlations were used to produce relative permeability curves as formulated in Equations 12 and 13.

12 $$k_{r} w = k_{r} wiro((S_{w} - S_{w} crit)/(1 - S_{w} crit - S_{o} irw))^{{N_{w} }}$$

13 $$k_{r} ow = k_{r} ocw((S_{o} - S_{o} rw)/(1 - S_{w} con - S_{o} rw))^{{N_{o} w}}$$

### Prediction of oil recovery from numerical simulations

**Fig. 13:**
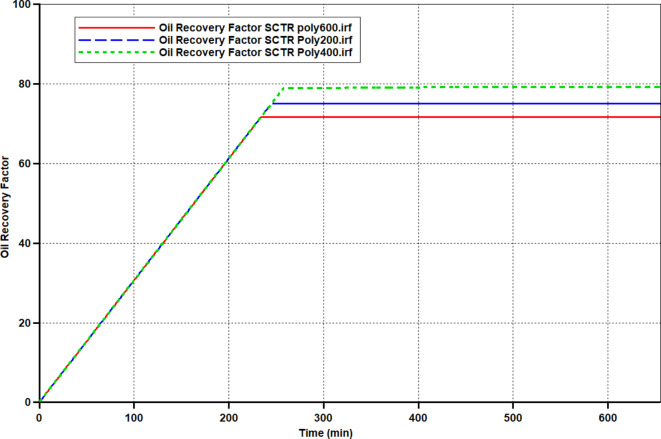
Oil recovery factor from numerical simulation (CMG STARS simulator) for Poly400, Poly200, and Poly 600.

After building models of polymer flooding at the core scale, the simulator was run to evaluate the oil recovery. The results of the oil recovery factor for the three EOR scenarios were presented using the CMG STAR (Figure 13). The recovery factor obtained by Poly 400, Poly 200_,_ and Poly 600 was 79.09%, 75.02%, and 71.66% respectively. The highest oil recovery was 79.09% (poly 400 flooding). A comparison of oil recovery by Poly 400, Poly 200, and Poly 600 in cases of flooding experiments and numerical simulation has been presented in Table 4. As detected, oil recovery by the simulation model matches the results of oil recovery by flood experiments. Furthermore, in consistency with the results of flooding experiments, the incremental recovery by the Poly 400 and Poly 200 solutions surpasses that of Poly 600. Consistent with the experimental flooding, this phenomenon is related to oil solubilization and improvement of displacing efficiency^60^.

**Table 4:** Oil recovery from simulation runs and compared with that of flooding experiments.

Run	Polymer solution	Total RF from flooding experiments (% OOIP)	Total RF from Simulation (% OOIP)
1	Poly 400	79	79.09
2	Poly 200	75	75.02
3	Poly 600	72	71.66

### Oil saturation profiles

**Fig. 14:**
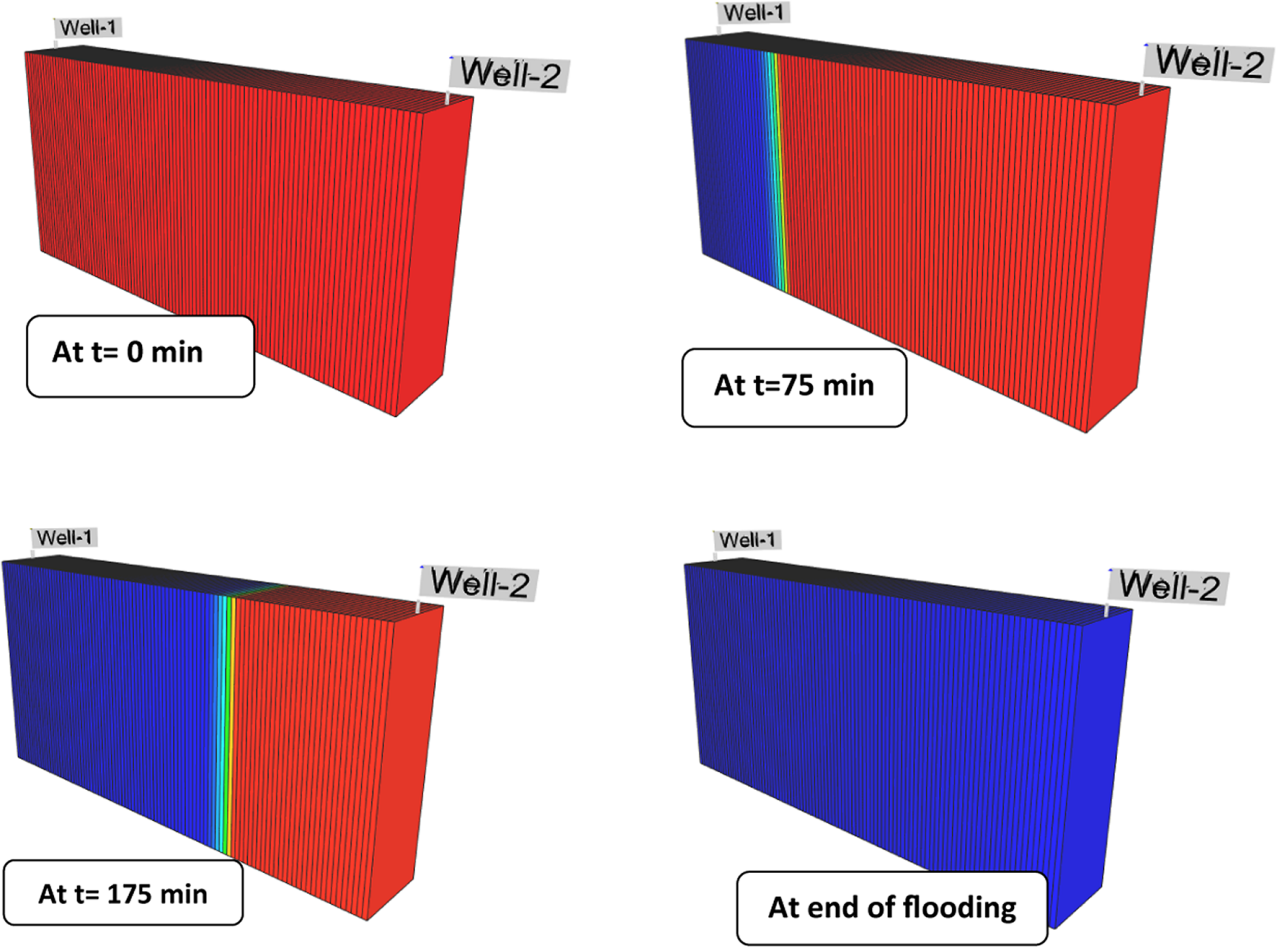
Oil saturation contours from CMG STARS simulator, showed by 3D Cartesian grids at different periods for the flooding of Poly 400.

**Fig. 15:**
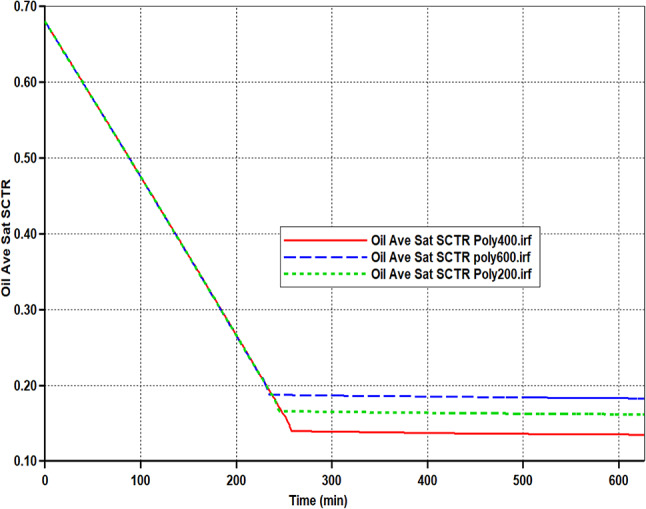
Oil saturation performance during Poly 400, Poly 200, and Poly 600.

Simulation of Core-scale displacement tests was built to qualitatively assess the secondary and tertiary recoveries. Based on the numerical simulation results, Figure 14 and Figure S2-S3 (Supplementary materials display maps of simulated oil saturation at various time intervals. At the initial time, the rock was saturated with oil. Typically, (soi) was measured as 68% for Poly 400, Poly 200, and Poly 600 and this corresponds to the initial oil in place. In each EOR scenario, an identical water solution was inoculated at (0.5 cc/min) as a procedure of secondary recovery. Throughout the water injection, the porous model’s oil saturation progressively dropped. As depicted in Figure 14 and Figure S2-S3 (Supplementary materials) the change in the color of the cartesian grid pictures from red to yellow or green implies a decrease in oil saturation. However, respective oil saturation reduction was also observed after the injection of poly 400. Nevertheless, it can be viewed that the oil-swept area of Poly 600 is smaller than other scenarios of Poly 400, and Poly 200 polymer flooding. The final residual oil saturation values corresponding to Poly 400, Poly 200, and Poly 600 were determined from history-match results as 13.5%, 16.1%, and 18.3%, respectively as presented in Figure 15 and Table 5. The results reveal that the lowest S_or_ recorded was for poly 400 flooding. The reduction of oil saturation for the poly 400 and poly 200 was significantly better than the poly 600 polymer flooding. Oil saturation mapping demonstrates that polymeric solutions improve the sweep efficiency of oil from the injection area to the production one^56^.

**Table 5:** Residual oil saturation (S_or_) after Poly 400, Poly 200, and Poly 600.

Polymer EOR scenario	Residual oil saturation (S_or_) after EOR scenarios
Poly 400	0.135
Poly 200	0.161
Poly 600	0.183

## Advantages, disadvantages, and cost analysis of fluorescent polymer microspheres used in EOR applications

Fluorescent polymer microspheres used in enhanced oil recovery applications offer several promising advantages and potential disadvantages that need to be considered, as shown in Table 6. From an economic perspective, the main goals of enhanced oil recovery projects are to maximize the recoverable oil and, consequently, the overall revenue generated by the oilfield. Typically, there are three main categories of expenses to consider during the execution of polymer flooding EOR: (a) expenses related to experimental research and simulation, (b) costs associated with project planning, construction, installation, and the initiation of injection operations, and (c) expenditures on chemicals. Nonetheless, a significant portion of the overall cost of polymer flooding is linked to chemical injections, constituting approximately 75% of the total expenses incurred in the polymer flooding process^61^. The production cost of fluorescent polymer microspheres involves specialized materials and processes, contributing to higher production costs. Moreover, Injecting and monitoring these microspheres in the reservoir requires additional equipment and operational expenses, further increasing the overall cost. On the other hand, the enhanced sweep efficiency and selective placement offered by fluorescent polymer microspheres can lead to a significant increase in oil recovery rates, potentially outweighing the initial costs. The injection of fluorescent polymer microspheres presents a promising approach to minimizing water production in oil reservoirs by improving sweep efficiency, controlling fluid flow, and selectively plugging water-producing zones, resulting in enhanced O/W ratios, and increased profitability. Furthermore, real-time monitoring facilitated by the fluorescence of these microspheres can aid in optimizing reservoir management strategies, potentially maximizing long-term production.

**Table 6:** The promising advantages and the potential disadvantages of fluorescent polymer microspheres used in EOR applications.

Advantages	Disadvantages
I. Fluorescent polymer microspheres can be modified to specific sizes and densities, allowing for precise control over their placement in oil reservoirs (Selective Placement)^3^ II. Microspheres can improve sweep efficiency by diverting injection fluids into un-swept zones, thereby increasing oil recovery rates^62^ III. Used to reduce water production by blocking water channels and thus reduce unwanted water production, leading to higher oil-to-water ratios^63^ IV. Fluorescent polymer microspheres are compatible with various reservoir fluids and can withstand high temperatures and pressures commonly encountered in oil reservoirs^3^ V. The fluorescence of these microspheres enables real-time tracking and monitoring of their movement within the reservoir, aiding in assessing their effectiveness^6^	I. Possible dye leakage which constraints the fields of application^3^ II. The cost and deployment of fluorescent polymer microspheres can be costly, especially for large-scale reservoir applications. Implementing this technology may require specialized expertise and equipment for manufacturing and injection, adding complexity to the EOR process^64^

## Environmental impact of fluorescent polymers in enhanced oil recovery

Recent studies have focused on developing more environmentally friendly fluorescent polymers for EOR applications^65^. For instance, some studies have explored the use of bio-based monomers and biodegradable components in the synthesis of fluorescent polymer microspheres. These approaches aim to reduce the environmental footprint of EOR operations while maintaining the desired functionality of the polymers^66^. The biodegradability of fluorescent polymers used in EOR can vary significantly depending on their chemical composition. Some researchers have reported success in natural and biodegradable surfactants in EOR^67^. Environmental concerns related to the use of fluorescent polymers in EOR include potential accumulation in soil and water systems and their impact on aquatic ecosystems. To address these issues, fluorescent carbon dots with high water solubility, biocompatibility, and nontoxicity stabilized foam for EOR have been developed^68^. Notably, the environmental friendliness of fluorescent polymers in EOR extends beyond their biodegradability. The ability of these materials to improve oil recovery efficiency can lead to reduced overall environmental impact by maximizing resource extraction from existing wells and potentially reducing the need for new drilling operations. The current study has not established the environmental impact and toxicity profile of the newly synthesized polymeric surfactant. Our primary focus in this work was on the synthesis and characterization of the surfactant’s performance in enhanced oil recovery applications. However, we fully understand the critical importance of ensuring that any materials used in oil recovery processes are environmentally safe and non-toxic. In our next work, we will take into consideration the toxicity and biodegradability tests of the polymeric surfactant. To sum up, compliance with environmental regulations is essential to ensure the safe use and disposal of fluorescent polymers in oil recovery operations, minimizing their impact on the environment. Emphasizing sustainable practices in the development and deployment of fluorescent polymers can help alleviate environmental risks and promote responsible resource management.

## The technical challenges and limitations

One of the main obstacles in utilizing fluorescent polymer microspheres for EOR is maintaining their stability in extreme reservoir environments. High temperatures, elevated pressures, and the presence of various ions in the formation water can compromise the fluorescent properties and structural integrity of the microspheres. To combat this issue, researchers are developing more thermally stable nanocomposite-preformed particle gels (PPGs)^69^. Another significant challenge lies in managing the size distribution and swelling behavior of fluorescent polymer microspheres within porous media. Inconsistent size distribution can result in uneven plugging and reduced effectiveness in profile control. Recent research by Zhou et al. (2023) has addressed this problem by creating temperature-sensitive P(NIPAM-AM) nano-microspheres with a narrow size distribution, demonstrating improved conformance control in heterogeneous reservoirs^70^. Furthermore, incorporating stimuli-responsive elements into microsphere design has shown promise in enhancing control over swelling behavior in different reservoir zones. For example, Jamali et al. (2020) developed pH-sensitive poly(acrylamide-co-methylenebisacrylamide-co-acrylic acid) hydrogel microspheres, offering better adaptability to varying reservoir conditions^71^. Ensuring that fluorescent polymer microspheres penetrate deep into the reservoir while maintaining their plugging efficiency is crucial for successful profile control. Khalil et al. (2021) tackled this challenge by developing thermosensitive core–shell Fe3O4@poly(N-isopropylacrylamide) nanogels. These nanogels exhibited improved migration through porous media due to their small size and reversible swelling behavior, with the added benefit of potential retrieval thanks to their magnetic core ^72^. The economic viability of fluorescent polymer microsphere technology compared to conventional EOR methods remains a hurdle for widespread adoption. Current research efforts are focused on optimizing synthesis methods and exploring more economical fluorescent monomers. Zhang et al. have made progress in this area by developing high-strength, self-degradable sodium alginate/polyacrylamide preformed particle gels, offering a potentially more cost-effective alternative to traditional polymer microspheres while maintaining good profile control properties ^73^. Practically, overcoming the technical challenges and limitations associated with fluorescent polymer microsphere technology in EOR requires a multidisciplinary approach involving chemistry, materials science, and reservoir engineering. Researchers and industry experts can enhance the efficiency of this technology by addressing issues related to penetration depth, retention, compatibility, reservoir complexity, monitoring, and cost as shown in Table 7. Continuous innovation, collaborative research efforts, and field trials are crucial for developing sustainable solutions that maximize the benefits of fluorescent polymer microspheres while mitigating technical constraints.

**Table 7:** The technical challenges and proposed solutions associated with fluorescent polymer microsphere technology in enhanced oil recovery.

Technical challenges	Proposed solutions
I. Fluorescent polymer microspheres may experience limited penetration into certain reservoir formations, reducing their effectiveness in targeting specific zones	• Engineering microspheres with optimized sizes and densities for better penetration • Utilizing surfactants or additives to enhance the mobility of microspheres in challenging reservoir conditions
II. Microspheres can face challenges in retention within the reservoir, leading to premature leakage and reduced efficiency	• Designing microspheres with surface modifications to improve retention • Developing encapsulation technologies to protect microspheres from premature degradation
III. Ensuring compatibility of fluorescent polymer microspheres with reservoir fluids and conditions to prevent adverse interactions	• Conducting thorough compatibility tests before deployment • Modifying polymer formulations for better stability under varying reservoir conditions
IV. Real-time monitoring and tracking of fluorescent polymer microspheres throughout the reservoir can be technically challenging	• Implementing advanced monitoring technologies, such as downhole sensors or tracers
V. Scaling up the production and deployment of fluorescent polymer microspheres for large EOR applications can be cost-prohibitive	• Developing cost-effective manufacturing processes for mass production • Conducting pilot studies to optimize deployment strategies and minimize costs

## Conclusion

The comprehensive experimental and theoretical studies outlined in this manuscript underscore the potential of cationic-based fluorescent-tagged polyacrylate copolymers as innovative agents for Enhanced Oil Recovery (EOR). Through meticulous synthesis, detailed characterization, and rigorously conducted flooding experiments complemented by simulation studies, this research demonstrates that these polymers significantly enhance oil recovery efficiency. The experimental results reveal that the incorporation of fluorescent-tagged polymers not only allows for the real-time monitoring and management of flooding processes but also effectively targets and mitigates the challenges posed by high-permeability layers in oil reservoirs. The ability of these polymers to adapt to various reservoir conditions, coupled with their enhanced plugging capabilities, facilitates improved sweep efficiency, thereby maximizing oil recovery. Flooding tests by fluorescent polymer microspheres achieved incremental oil recoveries (EOR) in the range of 6.1–13.1% of OOIP. Simulation models further validate the experimental findings, offering a predictive outlook of the polymers’ behavior in simulated reservoir conditions. These models are crucial for planning and optimizing EOR strategies, as they provide insights into the complex interactions between the polymers and reservoir rock and fluids. By improving the efficiency of oil recovery processes and offering a tool for enhanced reservoir management, these polymers represent a significant step forward in the sustainable exploitation of oil resources. Future work should focus on scaling these findings to field applications, and further refining their properties to enhance their applicability under diverse geological conditions. Moreover, Future research directions could encompass the following key areas:

- Developing stimuli-responsive fluorescent polymer microspheres that can undergo controlled changes in properties in response to reservoir conditions, such as temperature, or salinity variations in the reservoir.
- Explore the incorporation of nanomaterials into polymer microspheres to improve their stability, dispersibility, and interaction with reservoir fluids, leading to enhanced oil recovery performance.
- Develop advanced imaging and tracking techniques to monitor the movement and distribution of fluorescent polymer microspheres within the reservoir, enabling real-time visualization and optimization of oil recovery processes.
- Investigate the use of environmentally friendly and biocompatible materials in the design of fluorescent polymer microspheres to ensure minimal environmental impact and compatibility with reservoir conditions.

By pursuing these research directions, it may be possible to significantly enhance the effectiveness and applicability of fluorescent polymer microspheres in EOR. This could potentially lead to more efficient and sustainable oil recovery techniques across a wider range of reservoir types, ultimately improving the overall performance and economic viability of EOR operations.

## Electronic supplementary material

Below is the link to the electronic supplementary material.

Supplementary Material 1

## Data Availability

All data generated or analysed during this study are included in this published article [and its supplementary information files].

## Abbreviations

ATRP: Atom transfer radical polymerization
RAFT: Reversible addition-fragmentation chain transfer polymerization
FONs: Fluorescent organic nanoparticles
QDs: Carbon quantum dots
OOIP: Original Oil in Place
Q: Flow rate, cc/s
μ: Brine viscosity, cp
L: Model length, cm
Δp: The pressure difference, atm
A: Cross-section area, cm^2^
k: Absolute permeability
*kr*_*w*_: Water relative permeability for water-oil;
*k*_*row*_: Oil relative permeability for water-oil
*τ*: Shear stress (Pa)
*τ*_*0*_: Yield stress (Pa)
*K*: Flow consistency coefficient (Pa. s^-^^n^)
*γ*: Shear rate (s^–1^)
*n*: Flow behavior index (dimensionless parameter) that determines shear thinning nature
G′: Elastic modulus
G″: Viscous modulus
G*: Complex modulus
δ: Phase angle
(O/W): Oil-in-Water emulsions
Soi: Initial oil saturation
*S*_*wcon*_: Connate water saturation
*S*_*wcrit*_: Critical water saturation
*S*_*oirw*_: Irreducible oil saturation
*S*_*orw*_: Residual oil saturation
*Nw*: Water exponent
*N*_*ow*_: Oil-Water exponent

## References

[1] Liu, Y. & Zhang, P. Review of phosphorus-based polymers for mineral scale and corrosion control in oilfield. *Polymers* **14**(13), 2673 (2022). 35808717 10.3390/polym14132673 PMC9268766

[2] Kang, W. et al. Polymer concentration detection method based on fluorescent polymer to evaluate its retention and percolation. *J. Appl. Polym. Sci.* **136**(19), 47468 (2019).

[3] Shagymgereyeva, S., Sarsenbekuly, B., Kang, W., Yang, H. & Turtabayev, S. Advances of polymer microspheres and its applications for enhanced oil recovery. *Colloids and Surfaces B: Biointerfaces*. 113622 (2023). 10.1016/j.colsurfb.2023.113622 37931531

[4] Khattab, H., Gawish, A. A., Gomaa, S., Hamdy, A. & El-Hoshoudy, A. Assessment of modified chitosan composite in acidic reservoirs through pilot and field-scale simulation studies. *Sci. Rep.* **14**(1), 10634 (2024). 38724544 10.1038/s41598-024-60559-9 PMC11082220

[5] Khattab, H., Gawish, A. A., Hamdy, A., Gomaa, S. & El-hoshoudy, A. Assessment of a novel xanthan gum-based composite for oil recovery improvement at reservoir conditions; Assisted with simulation and economic studies. J. Polym. Environ. 1–29. (2024).

[6] Yang, H. et al. Influence mechanism of fluorescent monomer on the performance of polymer microspheres. *J. Mol. Liq.* **308**, 113081 (2020).

[7] Gao, Y., Zhang, J., Liang, J., Yuan, D. & Zhao, W. Research progress of poly (methyl methacrylate) microspheres: preparation, functionalization and application. *Eur. Polym. J.* **175**, 111379 (2022).

[8] Pak, Y. L., Wang, Y. & Xu, Q. Conjugated polymer based fluorescent probes for metal ions. *Coord. Chem. Rev.* **433**, 213745 (2021).

[9] Lu, X. et al. Novel fluorescent amphiphilic block copolymers: Controllable morphologies and size by self-assembly. *Eur. Polym. J.* **43**(7), 2891–2900 (2007).

[10] Banerjee, S. L., Hoskins, R., Swift, T., Rimmer, S. & Singha, N. K. A self-healable fluorescence active hydrogel based on ionic block copolymers prepared via ring opening polymerization and xanthate mediated RAFT polymerization. *Polym. Chem.* **9**(10), 1190–1205 (2018).

[11] Zhang, Z.-H. et al. A novel and modified fluorescent amphiphilic block copolymer simultaneously targeting to lysosomes and lipid droplets for cell imaging with large Stokes shift. *Eur. Polym. J.* **166**, 111030 (2022).

[12] Bou, S., Klymchenko, A. S. & Collot, M. Fluorescent labeling of biocompatible block copolymers: Synthetic strategies and applications in bioimaging. *Mater. Adv.* **2**(10), 3213–3233 (2021). 34124681 10.1039/d1ma00110h PMC8142673

[13] Wang, H. et al. Synthesis of fluorescent-tagged scale inhibitor and evaluation of its calcium carbonate precipitation performance. *Desalination* **340**, 1–10 (2014).

[14] Chen, H. et al. Amphiphilic copolymer fluorescent probe for mitochondrial viscosity detection and its application in living cells. *Spectrochim. Acta Part A Mol. Biomol. Spectrosc.* **252**, 119499 (2021). 10.1016/j.saa.2021.119499 33556793

[15] Li, M. et al. Self-assembly of pH-responsive and fluorescent comb-like amphiphilic copolymers in aqueous media. *Polymer* **51**(15), 3377–3386 (2010).

[16] Huang, Z. et al. One-pot synthesis and biological imaging application of an amphiphilic fluorescent copolymer via a combination of RAFT polymerization and Schiff base reaction. *Polym. Chem.* **6**(11), 2133–2138 (2015).

[17] Huang, Z. et al. Amphiphilic fluorescent copolymers via one-pot combination of chemoenzymatic transesterification and RAFT polymerization: synthesis, self-assembly and cell imaging. *Polym. Chem.* **6**(4), 607–612 (2015).

[18] Ji, X. et al. Facile construction of fluorescent polymeric aggregates with various morphologies by self-assembly of supramolecular amphiphilic graft copolymers. *Polym. Chem.* **6**(28), 5021–5025 (2015).

[19] Oshchepkov, M. et al. Synthesis and visualization of a novel fluorescent-tagged polymeric antiscalant during gypsum crystallization in combination with bisphosphonate fluorophore. *Crystals* **10**(11), 992 (2020).

[20] Li, Z. et al. Stability mechanism of O/W Pickering emulsions stabilized with regenerated cellulose. *Carbohydr. Polym.* **181**, 224–233 (2018). 29253967 10.1016/j.carbpol.2017.10.080

[21] El-Hoshoudy, A. Investigating the potential antiviral activity drugs against SARS-CoV-2 by molecular docking simulation. *J. Mol. Liq.* **318**, 113968 (2020). 32839634 10.1016/j.molliq.2020.113968 PMC7399655

[22] El-Hoshoudy, A. et al. New correlations for prediction of viscosity and density of Egyptian oil reservoirs. *Fuel* **112**, 277–282 (2013).

[23] El-hoshoudy, A., Matallah, M., Gouzi, H., Saidat, B., Khane, Y., Chabani, M. et al. Bioremoval of lead ion from the aquatic environment using lignocellulosic (Zea mays), thermodynamics modeling, and MC simulation. Int. J. Environ. Sci. Technol. 1–18. (2024).

[24] Ali, H. R., Mostafa, H. Y., Husien, S. & El-hoshoudy, A. Adsorption of BTX from produced water by using ultrasound-assisted combined multi-template imprinted polymer (MIPs); factorial design, isothermal kinetics, and Monte Carlo simulation studies. *J. Mol. Liq.* **370**, 121079 (2023).

[25] Salem, K. G., Tantawy, M. A., Gawish, A. A., Gomaa, S. & El-hoshoudy, A. Nanoparticles assisted polymer flooding: Comprehensive assessment and empirical correlation. *Geoenergy Sci. Eng.* **226**, 211753 (2023).

[26] El-Hoshoudy, A. Quaternary ammonium based surfmer-co-acrylamide polymers for altering carbonate rock wettability during water flooding. *J. Mol. Liq.* **250**, 35–43 (2018).

[27] El-Hoshoudy, A., Mohammedy, M., Ramzi, M., Desouky, S. & Attia, A. Experimental, modeling and simulation investigations of a novel surfmer-co-poly acrylates crosslinked hydrogels for water shut-off and improved oil recovery. *J. Mol. Liq.* **277**, 142–156 (2019).

[28] Wei, Q., Wang, Y., Zhang, Y. & Chen, X. Aggregation behavior of nano-silica in polyvinyl alcohol/polyacrylamide hydrogels based on dissipative particle dynamics. *Polymers* **9**(11), 611 (2017). 30965914 10.3390/polym9110611 PMC6418808

[29] Chhabra, R. P & Richardson, J. F. Non-Newtonian Flow: Fundamentals and Engineering Applications. Elsevier. (1999).

[30] Angar, N.-E. & Aliouche, D. Rheological behavior and reversible swelling of pH sensitive poly (acrylamide-co-itaconic acid) hydrogels. *Polym. Sci. Ser. A* **58**(4), 541–549 (2016).

[31] El-hoshoudy, A., Desouky, S., Betiha, M. & Alsabagh, A. Use of 1-vinyl imidazole based surfmers for preparation of polyacrylamide–SiO 2 nanocomposite through aza-Michael addition copolymerization reaction for rock wettability alteration. *Fuel* **170**, 161–175 (2016).

[32] Zhang, D. L., Liu, S., Puerto, M., Miller, C. A. & Hirasaki, G. J. Wettability alteration and spontaneous imbibition in oil-wet carbonate formations. *J. Pet. Sci. Eng.* **52**(1–4), 213–226 (2006).

[33] Barnes, H. A., Hutton, J. F., & Walters, K. An Introduction to Rheology. Elsevier. (1989).

[34] Zhong, H., He, Y., Yang, E., Bi, Y. & Yang, T. Modeling of microflow during viscoelastic polymer flooding in heterogenous reservoirs of Daqing Oilfield. *J. Pet. Sci. Eng.* **210**, 110091 (2022).

[35] Xie, F., Halley, P. J. & Avérous, L. Rheology to understand and optimize processibility, structures and properties of starch polymeric materials. *Progr. Polym. Sci.* **37**(4), 595–623 (2012).

[36] Salem, K. G., Salem, A. M., Tantawy, M. A., Gawish, A. A., Gomaa, S. & El-hoshoudy, A. A comprehensive investigation of nanocomposite polymer flooding at reservoir conditions: New insights into enhanced oil recovery. *J. Polym. Environ*. 1–21. (2024).

[37] Qiao, R. & Zhu, W. Evaluation of modified cationic starch for impeding polymer channeling and in-depth profile control after polymer flooding. *J. Ind. Eng. Chem.* **16**(2), 278–282 (2010).

[38] Koohi, A. D., Seftie, M. V., Ghalam, A., Moghadam, A. M. & Sabet, S. Z. Rheological characteristics of sulphonated polyacrylamide/chromium triacetate hydrogels designed for water shut-off. *Iran. Polym. J.* **19**(10), 757–770 (2010).

[39] Singh, R. & Mahto, V. Preparation, characterization and coreflood investigation of polyacrylamide/clay nanocomposite hydrogel system for enhanced oil recovery. *J. Macromol. Sci. Part B* **55**(11), 1051–1067 (2016).

[40] El-Hoshoudy, A., Desouky, S., Betiha, M. & Alsabagh, A. Use of 1-vinyl imidazole based surfmers for preparation of polyacrylamide–SiO2 nanocomposite through aza-Michael addition copolymerization reaction for rock wettability alteration. *Fuel* **170**, 161–175 (2016).

[41] Dave, P. N. & Macwan, P. M. Effect of functionalized multiwalled carbon nanotubes on the mechanical, swelling and viscoelastic properties of gum ghatti-cl-poly (NIPAm) hydrogels. *New J. Chem.* **48**(20), 9249–9261 (2024).

[42] Miri, T. Viscosity and oscillatory rheology. Practical food rheology: An interpretive approach. 7–28. (2011).

[43] Adewunmi, A. A., Ismail, S. & Sultan, A. S. Investigation into the viscoelastic response at various gelation performance, thermal stability and swelling kinetics of fly ash reinforced polymer gels for water control in mature oilfields. *Asia-Pac. J. Chem. Eng.* **12**(1), 13–24 (2017).

[44] Teodorescu, M., Morariu, S., Bercea, M. & Săcărescu, L. Viscoelastic and structural properties of poly (vinyl alcohol)/poly (vinylpyrrolidone) hydrogels. *RSC Adv.* **6**(46), 39718–39727 (2016).

[45] Pu, J., Zhou, J., Chen, Y. & Bai, B. Development of thermotransformable controlled hydrogel for enhancing oil recovery. *Energy Fuels* **31**(12), 13600–13609 (2017).

[46] Tongwa, P. & Bai, B. Degradable nanocomposite preformed particle gel for chemical enhanced oil recovery applications. *J. Pet. Sci. Eng.* **124**, 35–45 (2014).

[47] Aalaie, J. & Rahmatpour, A. Preparation and swelling behavior of partially hydrolyzed polyacrylamide nanocomposite hydrogels in electrolyte solutions. *J. Macromol. Sci. Part B* **47**(1), 98–108 (2007).

[48] Bai, M., Zhang, Z., Cui, X. & Song, K. Studies of injection parameters for chemical flooding in carbonate reservoirs. *Renew. Sustain. Energy Rev.* **75**, 1464–1471 (2017).

[49] Afolabi, F., Mahmood, S. M., Yekeen, N., Akbari, S. & Sharifigaliuk, H. Polymeric surfactants for enhanced oil recovery: A review of recent progress. *J. Pet. Sci. Eng.* **208**, 109358 (2022).

[50] El-Hoshoudy, A. N. Experimental and theoretical investigation of glycol-based hydrogels through waterflooding processes in oil reservoirs using molecular dynamics and dissipative particle dynamics simulation. *ACS Omega* **6**(45), 30224–30240 (2021). 34805657 10.1021/acsomega.1c01533 PMC8600538

[51] Abou-alfitooh, S. A., El-Hosiny, F. & El-hoshoudy, A. Experimental and computational study of modified biopolymer xanthan gum with synthetic vinyl monomers for enhanced oil recovery. *J. Polym. Environ.* 1–20. (2024).

[52] Hou, X. & Sheng, J. J. Properties, preparation, stability of nanoemulsions, their improving oil recovery mechanisms, and challenges for oil field applications—A critical review. *Geoenergy Sci. Eng.* **221**, 211360 (2023).

[53] Yang, H. et al. A novel active amphiphilic polymer for enhancing heavy oil recovery: Synthesis, characterization and mechanism. *J. Mol. Liq.* **391**, 123210 (2023).

[54] Kumar, A. & Mandal, A. Core-scale modelling and numerical simulation of zwitterionic surfactant flooding: designing of chemical slug for enhanced oil recovery. *J. Pet. Sci. Eng.* **192**, 107333 (2020).

[55] Goudarzi, A., Delshad, M. & Sepehrnoori, K. A critical assessment of several reservoir simulators for modeling chemical enhanced oil recovery processes. In *SPE reservoir simulation symposium.* OnePetro. (2013).

[56] Pal, N. & Mandal, A. Numerical simulation of enhanced oil recovery studies for aqueous gemini surfactant-polymer-nanoparticle systems. *AIChE J.* **66**(11), e17020 (2020).

[57] Khalilinezhad, S. S., Mohammadi, A. H., Hashemi, A. & Ghasemi, M. Rheological characteristics and flow dynamics of polymer nanohybrids in enhancing oil recovery from low permeable carbonate oil reservoirs. *J. Pet. Sci. Eng.* **197**, 107959 (2021).

[58] Saw, R. K., Singh, A., Maurya, N. K. & Mandal, A. A mechanistic study of low salinity water-based nanoparticle-polymer complex fluid for improved oil recovery in sandstone reservoirs. *Colloids Surf. A Physicochem. Eng. Aspects* **666**, 131308 (2023).

[59] Kumar, N., Pal, N. & Mandal, A. Nanoemulsion flooding for enhanced oil recovery: Theoretical concepts, numerical simulation and history match. *J. Pet. Sci. Eng.* **202**, 108579 (2021).

[60] Asl, F. O. et al. Impact of PAM-ZnO nanocomposite on oil recovery. *Fuel* **332**, 125941 (2023).

[61] Salem, K. G. et al. Key aspects of polymeric nanofluids as a new enhanced oil recovery approach: A comprehensive review. *Fuel* **368**, 131515 (2024).

[62] Cao, D., Han, M., Saleh, S., Ayirala, S. & Al-Yousef, A. SmartWater synergy with microsphere injection for permeable carbonates. In *SPE Middle East Oil and Gas Show and Conference.* D041S50R08 (SPE, 2021).

[63] Yu, X. et al. Degradable cross-linked polymeric microsphere for enhanced oil recovery applications. *RSC Adv.* **5**(77), 62752–62762 (2015).

[64] Lifton, V. A. Microfluidics: An enabling screening technology for enhanced oil recovery (EOR). *Lab Chip* **16**(10), 1777–1796 (2016). 27087065 10.1039/c6lc00318d

[65] Ahmadi, A. et al. Insight into nano-chemical enhanced oil recovery from carbonate reservoirs using environmentally friendly nanomaterials. *ACS omega* **7**(41), 36165–36174 (2022). 36278110 10.1021/acsomega.2c03076 PMC9583302

[66] Khan, M. Chemical and physical architecture of macromolecular gels for fracturing fluid applications in the oil and gas industry; current status, challenges, and prospects. *Gels* **10**(5), 338 (2024). 38786255 10.3390/gels10050338 PMC11121287

[67] Saha, R., Uppaluri, R. V. & Tiwari, P. Impact of natural surfactant (reetha), polymer (xanthan gum), and silica nanoparticles to enhance heavy crude oil recovery. *Energy Fuels* **33**(5), 4225–4236 (2019).

[68] Sakthivel, S., Adebayo, A. & Kanj, M. Y. Experimental evaluation of carbon dots stabilized foam for enhanced oil recovery. *Energy Fuels* **33**(10), 9629–9643 (2019).

[69] Durán-Valencia, C. et al. Development of enhanced nanocomposite preformed particle gels for conformance control in high-temperature and high-salinity oil reservoirs. *Polym. J.* **46**(5), 277–284 (2014).

[70] Zhou, Y. et al. Preparation and properties of temperature-sensitive P (NIPAM-AM) nano–microspheres in enhanced oil recovery. *Energy Fuels* **37**(1), 204–213 (2022).

[71] Jamali, A., Moghbeli, M., Ameli, F., Roayaie, E. & Karambeigi, M. Synthesis and characterization of pH-sensitive poly (acrylamide-co-methylenebisacrylamide-co-acrylic acid) hydrogel microspheres containing silica nanoparticles: Application in enhanced oil recovery processes. *J. Appl. Polym. Sci.* **137**(12), 48491 (2020).

[72] Khalil, M., Fahmi, A., Nizardo, N. M., Amir, Z. & Mohamed, J. B. Thermosensitive core–shell Fe3O4@ poly (N-isopropylacrylamide) nanogels for enhanced oil recovery. *Langmuir* **37**(29), 8855–8865 (2021). 34242029 10.1021/acs.langmuir.1c01271

[73] Zhang, X. et al. High-strength and self-degradable sodium alginate/polyacrylamide preformed particle gels for conformance control to enhance oil recovery. *Pet. Sci.* **19**(6), 3149–3158 (2022).

