# Supplementary material for: Experimental and theoretical investigation of cationic-based fluorescent-tagged polyacrylate copolymers for improving oil recovery
Source: Sci Rep. 2024 Nov 12;14:27689. doi: 10.1038/s41598-024-78128-5 (PMC11557845; doi:10.1038/s41598-024-78128-5)
Supplement: Supplementary file 1 — Supplementary Material 1 [file 41598_2024_78128_MOESM1_ESM.docx]

**Experimental and theoretical investigation of cationic-based fluorescent-tagged polyacrylate copolymers for improving oil recovery**

**Ali A. Abd-Elaal^1^, Salah M. Tawfik^1^, Ahmed Abd-Elhamid^1^, Khalaf G. Salem^2^, A.N. El-hoshoudy^3,4*^**

^1^Petrochemicals Department, Egyptian Petroleum Research Institute, Naser City, Cairo, Egypt.

^2^Department of Reservoir Engineering, South Valley Egyptian Petroleum Holding Company (GANOPE), Cairo, Egypt.

^3^PVT lab, Production Department, Egyptian Petroleum Research Institute, Naser City, Cairo, Egypt.

^4^PVT-Service Center, Production Department, Egyptian Petroleum Research Institute, Naser City, Cairo, Egypt.

Corresponding author e-mail: azizchemist@yahoo.com; abdelaziz.nasr@epri.sci.eg

Table S1. Summary of previous research related to fluorescent polymers.

| Author | Type | Method and applications |
| --- | --- | --- |
| Lu et al. (2007) ^1^ | Fluorescent amphiphilic copolymers | atom transfer radical polymerization (ATRP) method |
| Li et al. (2010) ^2^ | comb-like graft copolymers |  |
| Wang et al. (2014) ^13^ | A novel fluorescent-tagged scale inhibitor | to study the inhibition of scaling problems in cooling water systems |
| Huang et al. (2015) ^16^ | Fluorescent organic nanoparticles (FONs) | through RAFT polymerization and Schiff base combined |
| Banerjee et al. (2018) ^10^ | a self-healing hydrogel with fluorescence activity | by incorporating fluorescence-responsive ionic block copolymers (BCPs) |
| Kang et al. (2019) ^2^ | fluorescent polymeric materials | by copolymerizing acrylamide (AM) with the chemically modified rhodamine B for polymer flooding applications |
| Yang et al. (2020) ^6^ | specialized class of fluorescent microspheres | a novel approach for the conformance control process |
| Oshchepkov et al. (2020) ^19^ | preparation and characterization of a new fluorescent polyacrylate | scale inhibitor |
| Zhang et al. (2022) ^11^ | synthesized a fluorescent amphiphilic block polyacrylate copolymer | using RAFT polymerization aiming to advance research in biomedical fields |

| (a) |
| --- |
|  |
| (b) |
|  |
| (c) |
|  |
| Figure S1: Shear/Viscosity profile raw data for P200, 400, 600 Respectively |

|  |
| --- |

| 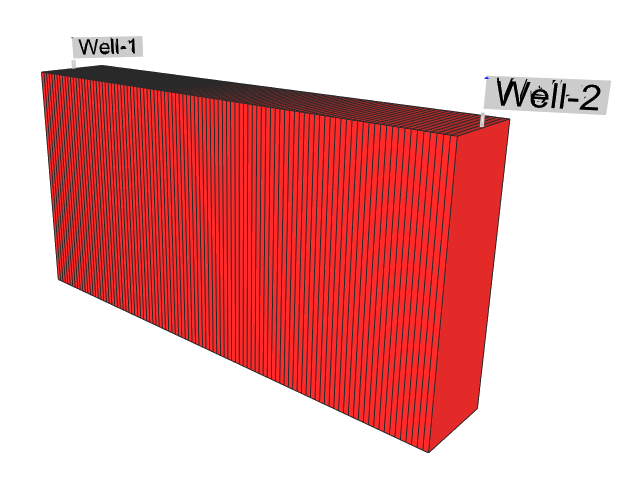  **At t= 0 min** | 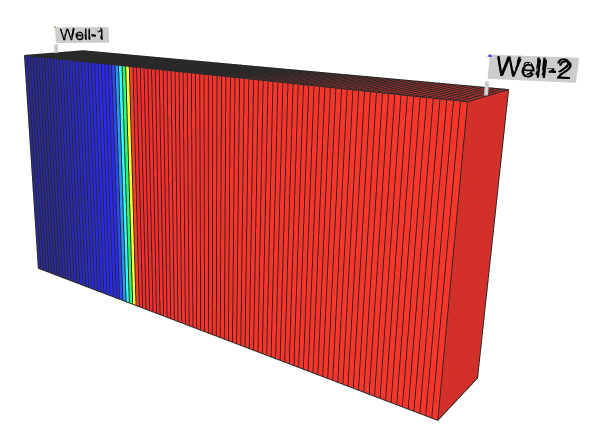  **At t=75 min** |
| --- | --- |
| 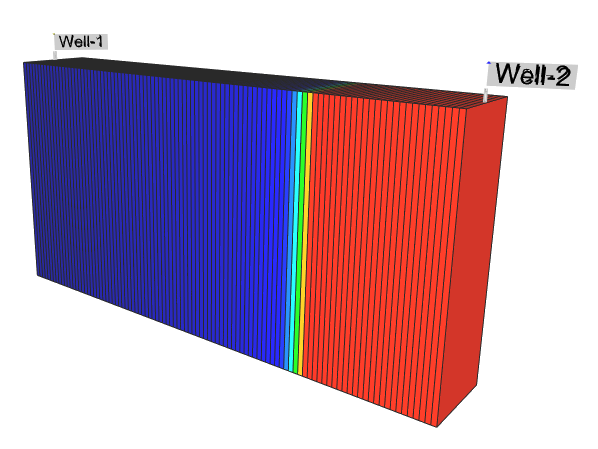  **At t= 175 min** | 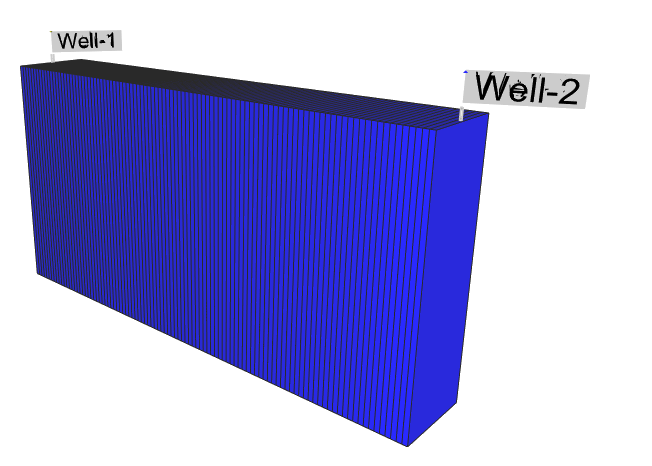  **At end of flooding** |
| Figure S2: Oil saturation contours from CMG STARS simulator, showed by 3D Cartesian grids at different periods for the flooding of Poly 200 | |

| 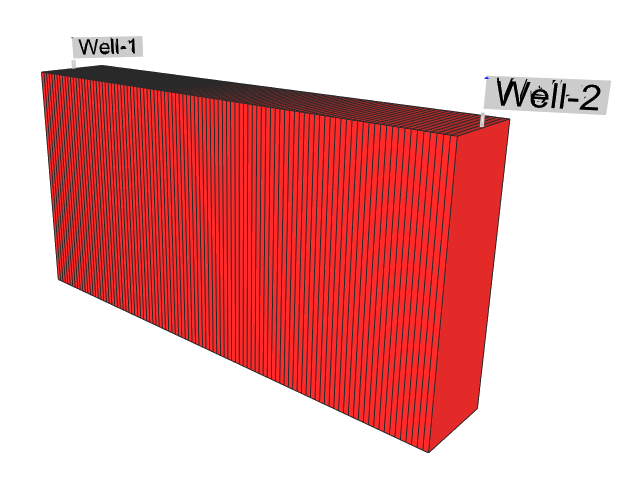  **At t= 0 min** | 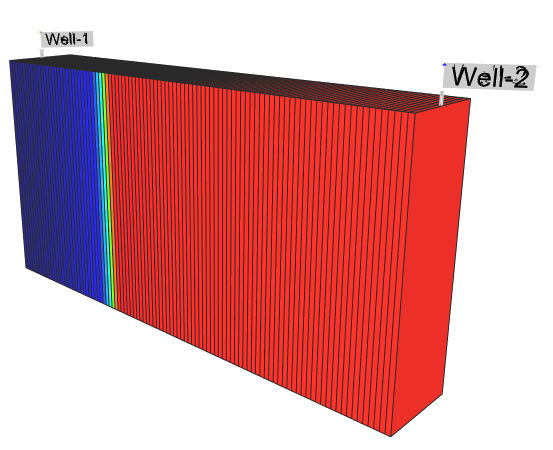  **At t=75 min** |
| --- | --- |
| 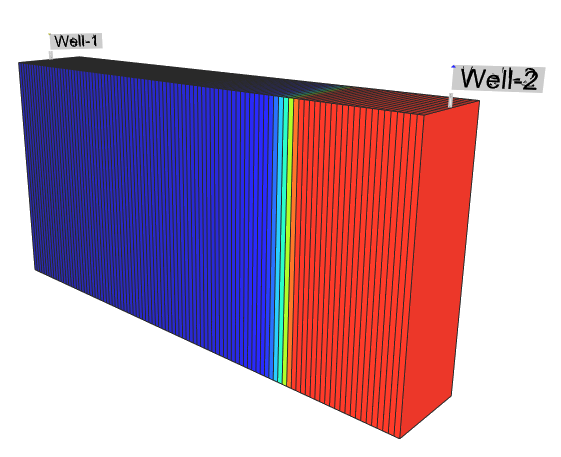  **At t= 175 min** | 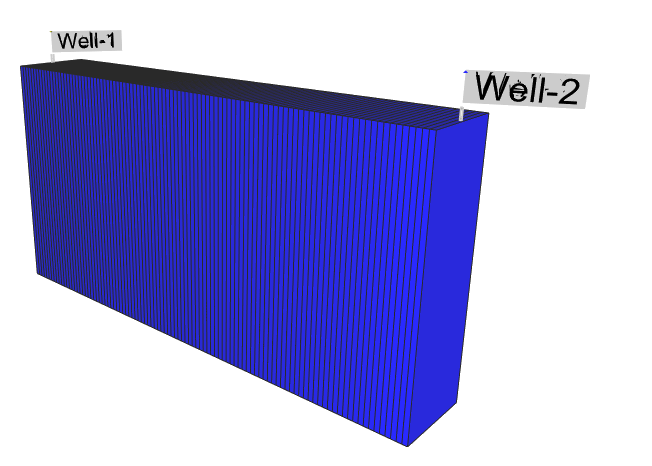  **At end of flooding** |
| Figure S3: Oil saturation contours from CMG STARS simulator, showed by 3D Cartesian grids at different periods for the flooding of Poly 600 | |
